# Genetic and antigenic evolution of H1 swine influenza A viruses isolated in Belgium and the Netherlands from 2014 through 2019

**DOI:** 10.1038/s41598-021-90512-z

**Published:** 2021-05-28

**Authors:** Sharon Chepkwony, Anna Parys, Elien Vandoorn, Wojciech Stadejek, Jiexiong Xie, Jacqueline King, Annika Graaf, Anne Pohlmann, Martin Beer, Timm Harder, Kristien Van Reeth

**Affiliations:** 1 Laboratory of Virology, Department of Virology, Parasitology and Immunology, Faculty of Veterinary Medicine, Ghent University, Salisburylaan 133, 9820 Merelbeke, Belgium; 2 Institute of Diagnostic Virology, Friedrich‐Loeffler‐Institut (FLI), Suedufer 10, 17493 Greifswald-Insel Riems, Germany

**Keywords:** Evolution, Genetics, Diseases

## Abstract

Surveillance of swine influenza A viruses (swIAV) allows timely detection and identification of new variants with potential zoonotic risks. In this study, we aimed to identify swIAV subtypes that circulated in pigs in Belgium and the Netherlands between 2014 and 2019, and characterize their genetic and antigenic evolution. We subtyped all isolates and analyzed hemagglutinin sequences and hemagglutination inhibition assay data for H1 swIAV, which were the dominant HA subtype. We also analyzed whole genome sequences (WGS) of selected isolates. Out of 200 samples, 89 tested positive for swIAV. swIAV of H1N1, H1N2 and H3N2 subtypes were detected. Analysis of WGS of 18 H1 swIAV isolates revealed three newly emerged genotypes. The European avian-like H1 swIAV (lineage 1C) were predominant and accounted for 47.2% of the total isolates. They were shown to evolve faster than the European human-like H1 (1B lineage) swIAV, which represented 27% of the isolates. The 2009 pandemic H1 swIAV (lineage 1A) accounted for only 5.6% of the isolates and showed divergence from their precursor virus. These results point to the increasing divergence of swIAV and stress the need for continuous surveillance of swIAV.

## Introduction

Influenza A viruses of the H1N1, H1N2 and H3N2 subtypes circulate in pigs. Due to the geographical segregation of pig populations and multiple introductions of viruses from either humans or birds, swine influenza A viruses (swIAV) differ genetically and antigenically between continents and regions. The surface proteins of influenza viruses, the hemagglutinin (HA) and neuraminidase (NA), frequently undergo minor amino acid changes, or antigenic drift, resulting in the emergence of new variants^1^. Also, due to their segmented genomes, influenza viruses are capable of exchanging genome segments by genetic reassortment, a process that results in new virus strains. As a result, multiple lineages of swIAV circulate globally.

Four major lineages of swIAV with HA genes from distinct origins are enzootic in swine in Europe. Avian H1N1 viruses were introduced to swine in 1979. They are thus referred to as ‘avian-like’ H1N1 viruses^2,3^ and their HA genes belong to lineage 1C in the global H1 classification system^4^. H3N2 viruses emerged in 1984 after a reassortment between a human H3N2 virus related to A/Port Chalmers/1/1973 and the ‘avian-like’ swine H1N1 virus. This resulted in a ‘human-like’ H3N2 virus with internal genes from the ‘avian-like' H1N1 virus^5–7^. H1N2 viruses were first reported in 1994 and were reassortants with the HA gene of a human seasonal H1N1 virus that was circulating in the mid-1980s and all other genes from the human-like swine H3N2 virus. They are thus referred to as the ‘human-like’ H1N2 viruses^8^ and their HA belongs to lineage 1B^4^. The 2009 pandemic H1N1 viruses belong to the latest of the four major swIAV lineages in the European swine population. They are reassortants comprising of gene segments from three major swIAV lineages^9,10^. The HA segment was derived from the classical swine H1N1 lineage, which has been present in North American swine since 1918, and belongs to lineage 1A^4^. The neuraminidase (NA) and matrix (M) genes were derived from the European ‘avian-like’ H1N1 virus, whereas the five remaining internal gene segments originated from a North American triple reassortant H3N2 swIAV.

Swine influenza A viruses that circulate in Europe are genetically and antigenically distinct from those circulating in other continents due to introductions from different origins and time points. In North America, five major lineages of swIAV circulate in pigs. The first lineage is the classical swine (lineage 1A) whose emergence coincided with the 1918 human pandemic that was caused by an H1N1 virus^11,12^. The second lineage emerged only in the late 1990s and consists of “triple” reassortant H3N2 viruses with surface proteins from a human H3N2 virus and internal protein genes from the latter virus, as well as classical swine and avian viruses. The classical swine lineage viruses reassorted with this H3N2 virus, leading to the diversification of their HA. This led to the emergence of the α, β, and γ clades within the classical swine H1 lineage. In the early 2000s, viruses containing HA and/or NA derived from human seasonal H1N1 influenza viruses were introduced to pigs resulting in the third lineage of swIAV. Two clades emerged from this human seasonal-like H1 (lineage 1B) swIAV, the δ1 and δ2 clades^13^. The 2009 pandemic H1N1 viruses formed the fourth lineage of swIAV^11,12^. The fifth lineage of swIAV emerged between 2010 and 2011 and it consists of viruses that acquired only the HA or both HA and NA from human H3N2 viruses that were circulating during this period. These are the so-called novel reassortant H3N2 viruses^14^. Currently, the predominant clades of H1 viruses in North America are the γ and δ1 clade viruses, which belong to lineages 1A and 1B respectively^4,15^. The situation is more complex in Asia, with lineages of swIAV circulating in Europe and North America also circulating in this continent^16,17^.

The four European lineages of swIAV have continued to evolve, either through further reassortment events or accumulation of mutations in their surface protein genes, resulting in the emergence of multiple genotypes and clades of influenza viruses. This prompted the formation of the then “European Surveillance Networks for Influenza in Pigs” (ESNIP 1, 2 and 3) between 2001 and 2013^18–21^. Between 2009 and 2013, 23 genotypes of swIAV were identified and 6 of them were responsible for 77% of the reported cases in 14 countries. A more recent surveillance study in 17 European countries from 2015–2018^22^ identified 16 more genotypes that had not been reported prior to the study. Notably, the 2009 pandemic H1N1 viruses were not detected in Belgium and the Netherlands, but reassortants containing their genes (HA and internal genes) were detected in the Netherlands^20–22^. The introduction of the 2009 H1N1 pandemic virus into the swine population has resulted in increased reassortment, leading to an explosion of novel genotypes of swIAV^13,23–25^.

There has been inconsistent surveillance of swIAV in swine populations worldwide, with uneven regional performance. Europe in particular, lags behind when compared to the USA. This could also be indicated partly by the differences in the amount of data from these two regions that are deposited in public sequence databases. In this study we aimed to: (1) Identify the subtypes and lineages of swIAV that circulated in swine in Belgium and the Netherlands from 2014 through 2019, (2) Determine the genotypes of the circulating viruses and explore possible introductions of genes from another continent, and (3) Genetically and antigenically characterize the predominant H1 swIAV subtype to better understand their evolutionary dynamics.

## Materials and methods

### Virus isolation and subtyping

Viruses were isolated from nasal swabs or lung tissues collected from pigs with influenza-like symptoms in Belgium and the Netherlands between 2014 and 2019. Virus isolation was done in Mardin-Darby Canine Kidney (MDCK) cells according to standard procedures^26^. Upon a first passage in MDCK cells, viral RNA was extracted using the INDICAL BIOSCIENCE IndiSpin pathogen kit according to the manufacturer’s instructions, and subtyped by multiplex RT-qPCR as previously described^27^.

### Whole genome sequencing

Extracted RNA was amplified using Invitrogen Superscript III One-Step RT-PCR with Platinum Taq (ThermoFisher Scientific, Waltham, USA) and influenza-specific primers^28^ designed to bind to the conserved 3’ and 5’ ends of all segments. PCR products were purified using AMPure XP Magnetic Beads (Beckman Coulter, Fullerton, USA) and quantified with the NanoDrop™ 1000 Spectrophotometer (ThermoFisher Scientific). The Rapid Barcoding Kit (RBK-004, Oxford Nanopore Technology, Oxford, UK) was used to prepare libraries for the MinION platform as per the manufacturer’s instructions. Samples were loaded onto a FLO-MIN106 R9.4.1 flow cell as per the manufacturer’s instructions and a five-hour run was conducted with standard settings. The MinIT (MinKNOW v19.12.1) was used to perform real time basecalling, and integrated Guppy v3.2.9 (ONT) was used to produce FastQ files. Mapping and final consensus production of the quality checked and trimmed reads was conducted in the Geneious Software Suite (v11.1.5; Biomatters) with Bowtie2 (v2.3.0 in the pre-set “Medium Sensitivity”), creating full genomes for all samples. Genotyping of the internal gene cassette was performed as previously described^22^.

### Genetic and antigenic characterization of the field isolates

We selected a total of 16 H1 swine and human influenza A viruses to use as reference viruses in the genetic and antigenic HA characterization of the isolates (Table 1). The viruses were selected to include historical and contemporary influenza strains that circulated in pigs in Europe and North America, as well as in humans. The European swIAV were selected to represent the European H1 avian-like (EU H1av; 1C) and the European H1 human-like (EU H1hu; 1B.1) lineages with emphasis on the past (1982–1994) and more recent (1998–2012) strains. The North American swIAV on the other hand were selected to represent clades that currently circulate in swine, known by their colloquial names as H1-γ (1A.3.3.3) and H1-δ1 (1B.2.2) clade viruses, which in this study are referred to as US H1-γ and US H1-δ1 clade viruses respectively. We also selected three human seasonal H1N1 viruses spanning 20 years of antigenic drift and two viruses representative of the 2009 H1N1 pandemic (H1pdm09) virus (1A.3.3.2).

**Table 1:** Reference influenza A virus strains used for genetic and antigenic characterization of the hemagglutinin (HA) of swIAV isolates.

Virus strain	Abbreviation	Subtype	Colloquial name of the H1 virus	H1 clade	GenBank HA accession no
A/swine/Finestère/2899/1982	swFI82	H1N1	EU H1av	1C.1	CY116348
A/swine/Belgium/1/1998	swBE98	H1N1	EU H1av	1C.2	FJ805962
A/swine/Gent/132/2005	swG05	H1N1	EU H1av	1C.2.1	CY116434
A/swine/Gent/28/2010	swG10	H1N1	EU H1av	1C.2.1	KP406525
A/swine/Scotland/410,440/1994	swSC94	H1N2	EU H1hu	1B.1	AF085413
A/swine/Gent/7625/1999	swG99	H1N2	EU H1hu	1B.1.2.1	AY590823
A/swine/Gent/177/2002	swG02	H1N2	EU H1hu	1B.1.2.1	CY116442
A/swine/Gent/26/2012	swG12	H1N2	EU H1hu	1B.1.2.1	KP406526
A/California/04/2009	CA09	H1N1	H1pdm09	1A.3.3.2	FJ966082
A/Slovenia/2903/2015	SL15	H1N1	H1pdm09	1A.3.3.2	EPI768541
A/swine/Ohio/511,445/2007	swOH07	H1N1	US H1γ clade	1A.3.3.3	EU604689
A/swine/Illinois/A0104020/2010	swIL10	H1N2	US H1δ1 clade	1B.2.2.2	JQ756323
A/swine/Alabama/A01104091/2016	swAL16	H1N2	US H1δ1 clade	1B.2.2.1	KX247675
A/Taiwan/1/1986	TW86	H1N1	Human seasonal H1	Other-human 1B.2	X17224
A/New Caledonia/20/1999	NC99	H1N1	Human seasonal H1	Other-human 1B.2	DQ508857
A/Brisbane/59/2007	BR07	H1N1	Human seasonal H1	Other-human 1B.2	CY058487

EU H1av—European avian-like H1; EU H1hu—European human-like H1; H1pdm09—2009 pandemic H1; US H1γ—United States of America H1 gamma clade; US H1δ1—United States of America H1 delta 1 clade.

### Genetic characterization of the HA of swIAV isolates

Full-length HA sequencing of virus isolates was partly performed at the Institute of Diagnostic Virology, Friedrich-Loeffler-Institut (FLI), Germany and partly at the Animal and Plant Health Agency (APHA), United Kingdom. The sequences were obtained by RT-qPCR as previously described^29^. QIAquick gel extraction kit (Qiagen, Hilden, Germany) was used to purify amplicons generated from a 1.5% agarose gel. The amplicons were then Sanger-sequenced using the RT-PCR primers. Analysis of the sequences was performed on an ABI 310 sequencer. The sequences were then curated using the Chromas Lite^@^ software http://www.technelysium.com.au/Chromas250Setup.exe) and assembled using CAP3 programme (http://doua.prabi.fr/software/cap3)^27^. The HA of the isolates were assigned to clades using the tool^4^ on the Influenza Research Database (IRD) at http://fludb.org. The H1 numbering scheme described by Burke and Smith was used^30^ To determine the genetic relationships between the virus isolates and the reference viruses, sequences were aligned with ClustalW and percentage amino acid (aa) identities in the HA1 and at antigenic sites^31,32^ determined using MEGA 7.0.26 software^33^.

All generated sequences were deposited in GenBank and assigned accession numbers MW362568-MW362741.

For phylogenetic analysis, maximum-likelihood trees were constructed with the IQ-TREE web server^34^. HA1 amino acid sequences from clades 1A, 1B and 1C of H1 influenza A viruses were aligned using the MAFFT web bioinformatics framework (https://mafft.cbrc.jp/alignment/software/)^35^. The constructed tree was presented and edited with FigTree V1.4.4 software (v1.4.4). The default Blosum62 substitution model was selected for the estimation of the amino acid substitution rates for both clade 1B and 1C viruses. Bayesian Markov chain Monte Carlo (MCMC) approach for Bayesian phylogenetic inference were performed with the BEAST package (version 1.10.4) using the strict molecular clock model. The model was run for more than 3,000,000 generations with every 2,000 cycles sampled for both clades. The fitting clock-rate and virus population evolutionary rate for the two clades of swIAV were analyzed with Tracer1.6 software (version 1.7.1). Effective sampling size (ESS > 200) of each run was obtained.

### Antigenic characterization of the HA of swIAV isolates

For antigenic characterization, virus isolates passaged twice on MDCK cells were tested for cross-reactivity with hyperimmune swine or ferret sera against the selected reference viruses (Table 1) in hemagglutination inhibition (HI) assays, according to standard procedures^26^. Briefly, sera were heat inactivated and then treated with receptor destroying enzyme (RDE) at 37 °C for 18 h to remove non-specific inhibitors. Non-specific agglutinins were removed prior to HI assay by adding 50% turkey red blood cells (TRBC). Tests were performed with two-fold serial dilutions of the sera, followed by incubation with 4 hemagglutinating units of virus for 1 h. TRBC at a concentration of 0.5% were added to the wells and incubated with the virus-serum mixtures for another 1 h. The reciprocal of the highest dilution of serum that completely inhibited hemagglutination was defined as the HI titer.

Using the HI data, a 3 dimensional (3D) antigenic map was constructed using the antigenic cartography software (https://acmacs-web.antigenic-cartography.org/). Antigenic distances were generated as raw data directly from the antigenic cartography software. The 3D cartographic projections were generated using ACMAS. Analysis of the table vs map distance was then conducted. Antigenic distances are measured in antigenic units (AU) and 1 AU is equivalent to a two-fold dilution in the HI assay.

The hyperimmune sera used in this study were produced against the selected reference viruses described and listed in Table 1. Different methods of production as well as different animal species (pigs and ferrets) were used. Antisera against the European H1 swIAV were produced in swine. For these, one influenza naïve pig per virus received two intramuscular vaccinations with ultra-violet light-inactivated monovalent whole virus vaccine (WIV) containing 256 hemagglutinating units (HAU) of virus in combination with an oil-in-water adjuvant (20% Emulsigen). The vaccines were administered 4-weeks apart. Blood was collected at the time of each vaccination and two weeks after the second vaccination, when a titer ≥ 160 was likely to have been achieved. Pigs were then humanely euthanised with sodium pentobarbital and large volumes of blood collected for serum. Antisera against the US H1γ and H1δ clade swIAV were generously provided by the United States Department of Agriculture (USDA). These were post-vaccination antisera that were produced in swine as described by Lewis et al.^36^. Antisera against the human viruses were generously provided by Dr. John McCauley of the Francis Crick Institute in London, United Kingdom. These were post-infection ferret sera.

The samples used in this study were obtained from pig holdings for routine veterinary diagnosis on respiratory diseases. Examination and reporting followed the standard business guidelines of Ghent University. No additional sampling was instigated on the basis of this study. Therefore, a separate ethical approval was not required.

## Results

### Subtyping of swIAV isolates

Out of the 200 samples that we received over the 6-year period, 89 tested positive for swIAV by virus isolation in MDCK cells (Table 2). Seventeen of these viruses were isolated from pigs in the Netherlands while the other 72 originated from pigs in Belgium. In total, there were 71 H1 and 18 H3 swIAV isolates. Based on subtyping results, viruses with the EU H1av HA accounted for 47.2% of the total positive isolates while those with the EU H1hu and H3 HA accounted for 27.0 and 20.2%, respectively. Viruses with the H1pdm09 HA accounted for only 5.6% of the total isolates. The most dominant subtype was H1N1 (40 out of 89 isolates) from the EU H1av lineage, followed by H1N2 (24 out of 89 isolates) from the EU H1hu lineage and H3N2 (18 out of 89 isolates) respectively. Only one isolate belonged to the 2009 pandemic H1N1 subtype. Six isolates were reassortants between the above-mentioned subtypes. Two of these were H1avN2, three were H1pdmN2 and one H1pdmN1 virus but with the NA derived from the EU H1av lineage. We could not identify two isolates by RT-qPCR due to insufficient RNA material. Based on cross-HI data (Supplementary Table S2 online) and neuraminidase inhibition (NI) assay (Supplementary Table S6 online), they were characterized as H1huN2 (A/swine/Gent/175/2014) and H3N2 (A/swine/Gent/34/2014). H3 isolates were not further analyzed in this study.

**Table 2:** Subtypes of swIAV isolated from pigs in Belgium and the Netherlands in the period 2014–2019.

Isolate subtype	Isolate lineage	Number of isolates per year						
		2014	2015	2016	2017	2018	2019	**Total**
H1N1	H1avN1av	9	3	1	4	9	14	**40**
	H1pdmN1av					1		**1**
	H1pdmN1pdm						1	**1**
H1N2	H1huN2	5		1	1	8	9	**24**
	H1avN2				1		1	**2**
	H1pdmN2	1					2	**3**
H3N2	H3huN2	8			2	4	4	**18**
Number of positive samples		23	3	2	8	22	31	**89**
Total number of samples		51	11	3	24	58	53	**200**

The three subtypes of swIAV that circulate in swine are shown in first column. Within each subtype, the lineages/origin (second column) of the HA and NA are indicated: av—European avian-like; hu—European human-like; pdm—2009 pandemic.

### Genotypic analysis

**Figure 1:**
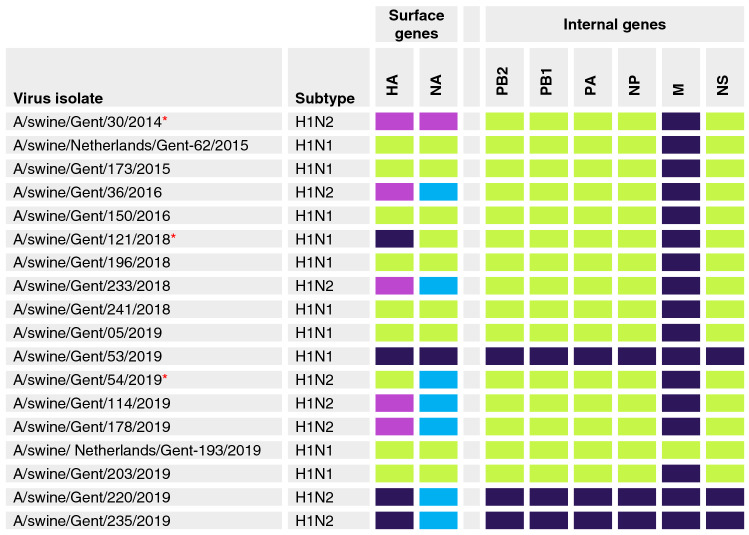
Genotypes of 18 H1N1 and H1N2 swIAV isolated in Belgium and the Netherlands between 2014 and 2019. The colour coding assigned to gene segments is based on their origin: green rectangle European avian-like H1N1, magenta rectangle European human-like H1N2, dark blue rectangle 2009 pandemic H1N1, light blue rectangle human-like H3N2. The N2 of the isolates originated either from the European human-like H1N2 lineage (swSC94-like; purple rectangle) or from the European human-like H3N2 lineage (swG84-like; light blue rectangle)^21^. The three viruses with new genotypes are shown with asterisks (*).

In total, 19 out of the 71 positive H1 isolates were selected for WGS. These included reassortant isolates as well as isolates that had a low signal in RT-qPCR. The isolates were selected for WGS on basis of subtype, date and location of origin so as to represent the full temporal, geographical and viral scope of the field sample collection. One sample, A/swine/Gent/55/2014, had low-coverage sequencing data and was therefore omitted from the analysis. Figure 1 shows genotypes of 18 H1 swIAV. As for the HA, these isolates belonged to different clades within the three H1 lineages: EU H1av (clade 1C.2.1 and 1C.2.2), EU H1hu (clade 1B.1.2.1) and H1pdm09 (clade 1A.3.3.2). Isolates of the H1N2 subtype had their NA derived from the prototype EU H1hu virus A/swine/Scotland/410,440/1994 (swSC94-like N2), or from the human-like H3N2 virus A/swine/Gent/1/1984 (swG84-like N2) as previously described^21^. Three out of the 18 sequenced isolates had genotypes (shown with red asterisk) that were not reported during the 2009–2013 influenza surveillance in Europe (Watson et al., 2015) and in the most recent surveillance study in Europe^22^. Interestingly, all the EU H1av and the EU H1hu viruses retained the internal genes of avian origin except for the M gene which, in 17 out of 18 viruses, was of H1pdm09 origin. The H1pdm09 viruses seem to have been circulating in pigs over the 6-year period although the first isolation of a wholly H1pdm09 virus in Belgium was in 2019.

### Genetic characterization of the HA of swIAV isolates

**Figure 2:**
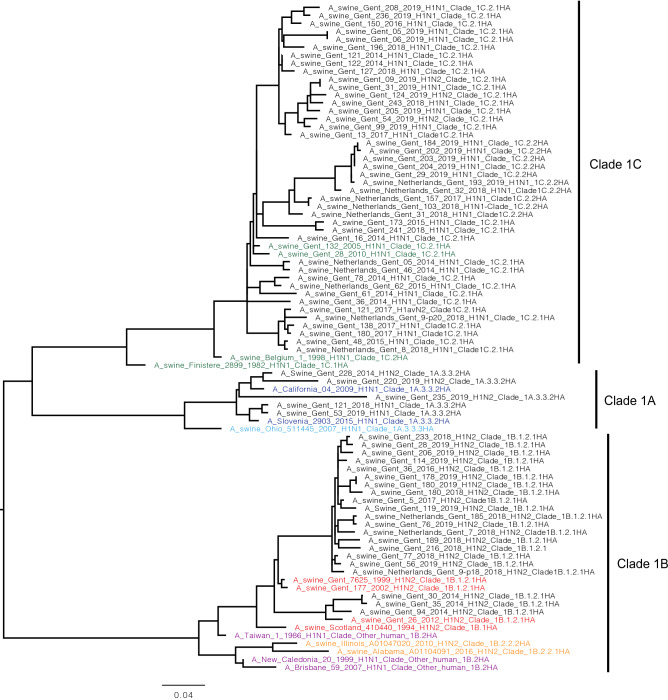
Phylogenetic tree of H1 swIAV isolates and reference viruses. HA1 amino acid sequences from clades 1A, 1B and 1C of H1 influenza A viruses were aligned using the MAFFT web bioinformatics framework^35^. The constructed tree was presented and edited with FigTree V1.4.4 software (v1.4.4). The names of the isolates are shown in black while those of the reference viruses are colored according to their lineages: EU H1av (green), EU H1hu (red), H1pdm09 (blue), US H1γ (light blue), US H1δ (orange), human H1 viruses (purple).

Due to sequencing failures (partial sequences), the HA sequences of three viruses (A/swine/Gent/55/2014, A/swine/Gent/93/2014, A/swine/Gent/175/2014) were not included in this analysis. We first identified the HA clade to which each isolate belonged (Supplementary Table S4 online). As observed in the genotypic analysis, the HA of the EU H1av (lineage 1C) isolates belonged to two clades: 1C.2.1 (32 isolates) and 1C.2.2 (10 isolates). On the other hand, the HA of all the EU H1hu (lineage 1B) swIAV isolates belonged to clade 1B.1.2.1 (21 isolates). The HA of the H1pdm09 (lineage 1A) swIAV isolates belonged to clade 1A.3.3.2 (5 isolates). To characterize the evolution of the isolates, we compared aa identities in the HA1 domain of the HA genes of 68 isolates with those of historical and contemporary swIAV circulating in pigs in Europe and North America as well as human influenza viruses. A phylogenetic tree based on the HA1 sequences of the isolates and the reference viruses is shown in Fig. 2.

The HA1 contains the known immunodominant epitopes and the receptor binding site^31^. The genetic relatedness of HA1 genes of the isolates and those of the reference viruses are shown in Supplementary Table S1 online. Among the EU H1av viruses, the highest genetic diversity in the HA1 was observed between the isolates of both clades 1C.2.1 and 1C.2.2, and clade 1C.1 reference virus (% aa identity ≤ 88.7). Percentage aa identities with reference viruses belonging to clade 1C.2.1 and clade 1C.2.2 were higher, i.e. between 92.0 and 96.8 for clade 1C.2.1 isolates and between 91.1 and 95.1 for clade 1C.2.2 isolates. The EU H1hu isolates (clade 1B.1.2.1) showed varying genetic relationships with the EU H1hu reference viruses of clades 1B.1 (89.2–91.1% aa identity) and 1B.1.2.1 (87.5–96.0% aa identity). As expected, they were divergent from the US H1δ clades 1B.2.2.2 (82.5–84.6% aa identity) and 1B.2.2.1 (80.4–82.5% aa identity) reference viruses. These viruses were also more divergent from the recent human seasonal viruses, NC99 and BR07 (82.8–87.4% aa identity) as compared with the past human seasonal virus, TW86 (89.0–90.5% aa identity). The human viruses (TW86, NC99, BR07) are classified as other-human 1B.2 according to the H1 classification system^4^. Due to its close genetic relationship with the EU H1hu viruses (1B.1.2.1), and also for clear comparisons in this study, we show TW86 as belonging to 1B.1-like clade. We consider TW86 as the ancestor virus of the EU H1hu viruses. As expected, the H1pdm09 isolates were more closely related to reference viruses of the same clade 1A.3.3.2 (88.7–97.2% aa identity) than to the US H1γ virus of clade 1A.3.3.3 (84.4–89.3% aa identity), which is their supposed precursor. However, out of the five H1pdm09 virus isolates, one strain was more divergent from reference viruses of the same clade. This virus shared only 88.7% aa identity with reference viruses, compared to 91.4–97.2% for the other four isolates. The H1 of all isolates were closely related to those of reference viruses of clades known to circulate in European swine, but they were distant from H1 of reference viruses representing clades that only circulate in North America. Thus, there was no indication of a spillover of North American swIAV strains into the European swine population.

We also examined the genetic relatedness at antigenic sites between the virus isolates and the reference viruses. The HA1 of H1 influenza viruses contains receptor binding sites surrounded by five antigenic sites—Sa, Sb, Ca1, Ca2 and Cb—which contain 50 aa in total^31,37^. We determined percentage aa identities among these 50 aa (Supplementary Table S5 online). We observed a similar trend of genetic relatedness as that in the aa of the entire HA1, but percentage aa identities at antigenic sites were way lower than in the HA1 (Supplementary Table S1 A-D online). This indicates that aa changes occur more frequently at antigenic sites.

We also evaluated the rate of aa substitution in the HA1 of H1 isolates. The substitution rates, as determined by BEAST analysis, suggest that the EU H1av swIAV evolve at a mean rate of 4.06 × 10^–3^ substitutions per site per year (95% highest posterior density (HPD): 3.18 × 10^–3^, 5.02 × 10^–3^), while the EU H1hu swIAV evolve at a mean rate of 3.76 × 10^–3^ substitutions per site per year (95% HPD: 3.02 × 10^–3^, 4.55 × 10^–3^). This indicates that the mean rate of evolution of the EU H1av viruses is faster than that of the EU H1hu viruses. We could not determine the substitution rates of the H1pdm09 swIAV isolates because there were only five isolates in this group of viruses.

### Antigenic characterization of the HA of swIAV isolates

**Figure 3:**
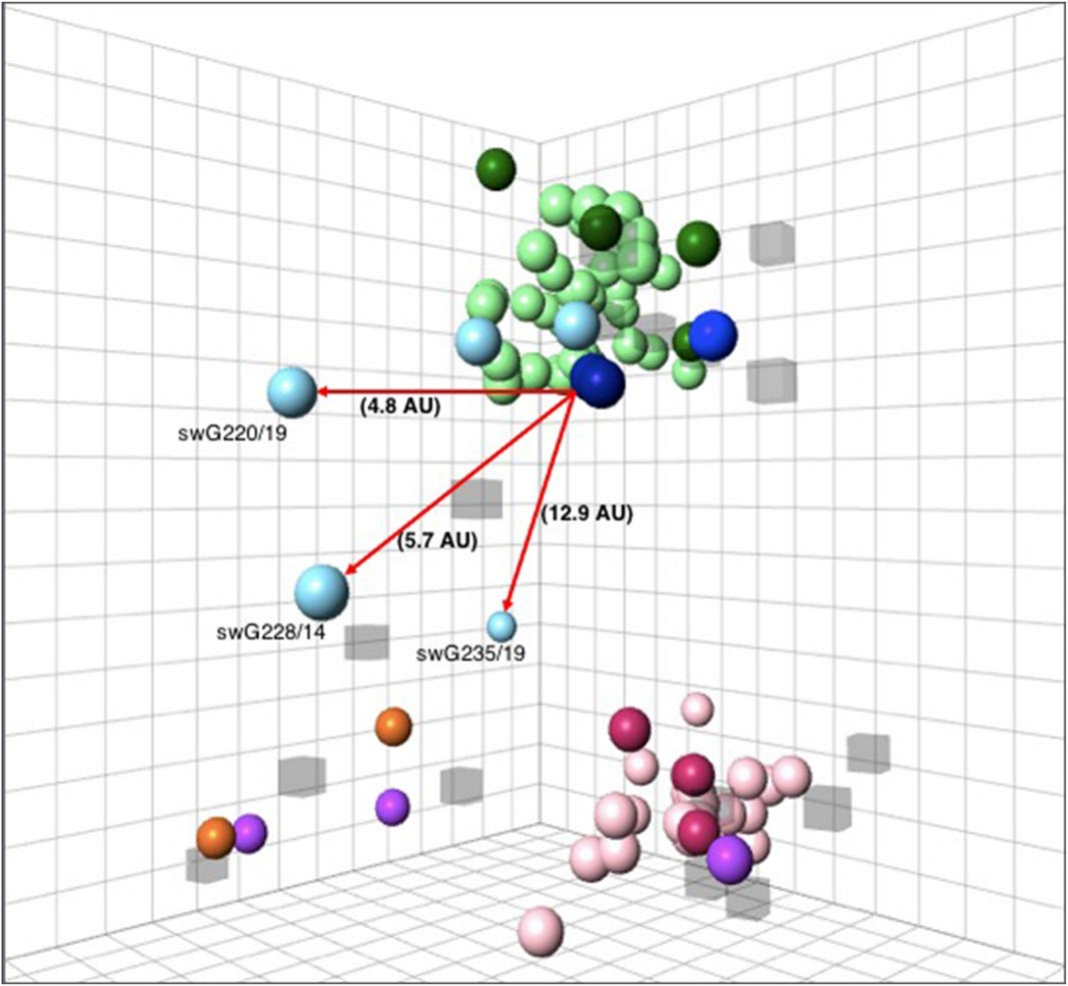
Antigenic diversity of H1 swIAV isolated in Belgium and the Netherlands in 2014–2019. A 3D map was constructed based on the reactivity of the isolates in HI assay, with sera against the reference viruses, which included: dark green sphere EU H1av, maroon sphere EU H1hu, dark blue sphere H1pdm09, blue sphere US H1γ, orange sphere US H1δ1 and purple sphere Human H1. The H1 isolates are colored using lighter shades of the color of the reference viruses belonging to the same or related lineage: light green sphere EU H1av, pink sphere EU H1hu and light blue sphere H1pdm09. The virus strains are shown as spheres while the respective antisera are shown as semi-transparent cubes. Antigenic distances of the three H1pdm09 outliers from the H1pdm09 reference virus (CA09) are shown with red arrows. The squares in the grid represent antigenic distances. One square is equal to a two-fold change in HI titer, which is equal to 1 antigenic unit (AU). Analysis of the table vs map distance was conducted generating a moderately high r^2^ score value (r^2^ = 0.71).

To determine antigenic relationships within and between H1 swIAV lineages, we performed HI assays with 71 H1 swIAV isolates and antisera against 16 historical and contemporary European and North American swIAV strains as well as human influenza A strains (Supplementary Table S2 online). There were 43 EU H1av isolates, 23 EU H1hu isolates and 5 H1pdm09 isolates. Using the obtained HI data, we generated a 3D antigenic map (Fig. 3). The swIAV isolates formed two antigenic clusters. The EU H1av isolates (light green spheres) clustered with EU H1av reference viruses (dark green spheres) as well as with the H1pdm09 (dark blue spheres) and US H1γ (blue spheres) reference viruses. On the other hand, the EU H1hu isolates (pink spheres) clustered with EU H1hu reference viruses (maroon spheres) and one human reference virus (purple sphere). All EU H1av isolates, regardless of the clade they belonged to, reacted with antisera against EU H1av reference viruses (HI titers ≥ 40). On the other hand, all EU H1hu isolates except for three isolates that failed to react with swSC94, reacted with antisera against EU H1hu reference viruses (HI titers ≥ 40). Low (HI titers ≤ 40) or no cross-reactivity was observed between viruses belonging to these two lineages. The EU H1av isolates also reacted with antisera against the H1pdm09 and the US H1γ reference viruses. However, all isolates (except 2) reacted with antisera against CA09 (H1pmd09) while only a few isolates, 12 and 7 out of 43, reacted with antisera against SL15 (H1pdm09) and OH07 (US H1γ) reference viruses respectively. There was low (HI titer ≤ 40) or no cross-reactivity between all the isolates and the US H1δ viruses (orange spheres) and human viruses (purple spheres) except for reactivity between the EU H1hu isolates and one past human virus, TW86 (HI titer ≥ 40 for 16 out of 23 isolates). Two of these isolates also had a titer of 80 against NC99.

Antigenic distances from the reference viruses ranged between 0.7 to 7.0 AU within EU H1av and 0.4 to 7.6 AU within EU H1hu isolates (Supplementary Table S3). On the other hand, antigenic distances within the H1pdm09 isolates ranged between 0.8 and 13.2 AU. Two out the five H1pdm09 isolates had antigenic distances ranging between 2.6 and 6.5 AU away from the EU H1av reference viruses, similar to what was observed within the EU H1av isolates. Their distances from the H1pdm09 reference viruses ranged between 0.8 and 2.1 AU. The other three were outliers (as shown on the map), with antigenic distances ranging between 6.7 and 13.2 AU from the EU H1av reference viruses and 4.8 to 13.3 from the H1pdm09 reference viruses.

Overall, all isolates were antigenically divergent from the US H1δ viruses and the recent human viruses (NC99 and BR07) as displayed on the map.

**Figure 4:**
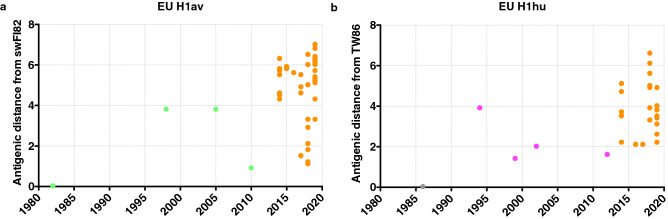
Antigenic distances of the EU H1av isolates (**a**) and EU H1hu isolates (**b**) from their ancestor viruses swFI82 and TW86 respectively. The virus isolates are shown in orange circles while the reference viruses are colored differently; green circle EU H1av, magenta circle EU H1hu and grey circle Human H1.

We retrieved antigenic distances from the antigenic map and examined distances of the EU H1av and EU H1hu isolates from their supposed ancestor viruses, swFI82 and TW86 respectively (Fig. 4). We observed more antigenic diversity from the ancestor virus within the EU H1av isolates (1.1 to 7.0 AU) as compared to the EU H1hu isolates (2.1 to 6.6 AU). The average evolution rates determined from antigenic distances between the ancestor viruses and viruses isolated in 2019, showed that the EU H1av isolates evolved at a rate of 0.15 AU per year while the EU H1hu isolates evolved at a rate of 0.10 AU per year. Taken together, these results suggest that the EU H1av viruses evolve at a slightly higher rate as compared to the EU H1hu viruses.

In order to explain the antigenic differences observed between viruses within the same lineage, we examined aa substitutions that have been previously considered important, and associated with antigenic changes^38–40^. These included aa substitutions R43L, F71I, K141E, E153K, E155G, N156D, D187N, S271P and a deletion at aa position 130 (Δ130). Three of these aa positions (43, 130, 271) are located outside the antigenic sites. Among the EU H1av viruses, substitution E155G (13 out of 42 isolates) was observed between EU H1av reference virus, swFI82, and the isolates (Table 3). Ten of these isolates had antigenic distances ranging between 5.0 and 6.8 AU away from swFI82. Distances between the other three isolates and swFI82 were < 5.0 AU. Interestingly, two out of these three also had another common substitution, L71I. In total, 11 EU H1av isolates had the L71I substitution. For the EU H1hu isolates, there were no aa substitutions at these putative important positions when compared to TW86 or the EU H1hu reference viruses expect for two viruses. One virus had an aa substitution at position 141, while the other virus had a substitution at position 153. On the other hand, aa substitutions R43L, I71F, E141K, N187D and S271P were observed between the EU H1hu isolates and the US H1δ reference viruses. The same substitutions were also observed with the recent human viruses NC99 and BR07. This could explain the antigenic differences observed between the EU H1hu isolates and US H1δ and the two more recent human viruses. Among the five H1pdm09 isolates, substitutions were observed in three of them: swG228/14 (E155G, D187S), swG220/19 (K43R, D187S) and swG235/19 (E155T, D187N). These three viruses were shown to be antigenically divergent from their 2009 precursor in the antigenic map. The most divergent H1pdm09 isolate, swG235/19, also had additional aa substitutions, N156D, which has been previously associated with antigenic changes^40^.

**Table 3:** Presence ( +) or absence (-) of important amino acid substitutions or deletion (Δ).

Substitution or deletion	Iso:	EU H1av	EU H1hu	H1pdm09	EU H1hu		
	Ref:	EU H1av	EU H1hu	H1pdm09	Past hu	Recent hu	US H1δ
R43L		–	–	–	–	+	+ /− (16)
F/L71I		+ /− *(11)	–	–	–	+ *	+ *
Δ130		–	–	–	–	–	–
K141E		–	+ /− (1)	–	+ /− (1)	+	+
E153K		–	+ /− (1)	–	+ /− (1)	–	–
E155G		+ /− (13)	–	+ /− (1)	–	–	–
N156D		–	–	+ /− (1)	–	–	–
D187N/S		–	–	+ /− (3)	–	+	+
S271P		–	–	+ /− (1)	–	+	+

Isolates of each lineage were compared with reference viruses belonging to the same lineage. Isolates belonging to the EU H1hu lineage were also compared with the US H1δ and past (TW86) and recent (NC99, BR07) human seasonal reference viruses due to their common origin. The symbol + /− is used where some isolates in the same lineage have the substitution while others do not. The number of isolates with the substitution are shown in brackets. In total, there were 42 EU H1av HA sequences, 21 EU H1hu and 5 H1pdm09 HA sequences.

Iso—isolates, Ref—reference viruses, hu—human viruses.

The asterisks denote a different substitution; L71L for EU H1av and I71F for the EU H1hu isolates when compared to the US H1δ and the recent human reference viruses.

## Discussion

We isolated 89 swIAV from pigs in Belgium and the Netherlands between the year 2014 and 2019, mainly H1 (71) and only a few H3 viruses (18). Because of the limited number of H3 viruses, we focused on the characterization of H1 swIAV isolates. Most of these (47.2%) had an HA derived from the EU H1av (1C) lineage and could be divided into two clades; 1C.2.1 and 1C.2.2. We isolated fewer EU H1hu (1B) lineage viruses (27%) all belonging to clade 1B.1.2.1. Only a few isolates (5.6%) had an HA derived from the H1pdm09 (1A) lineage and they all belonged to clade 1A.3.3.2.

For the first time, we isolated viruses with HA derived from the H1pdm09 lineage from pigs in Belgium. In the Netherlands, however, reassortant viruses containing all genes except the NA from the H1pdm09 viruses have been reported since 2012^21,22^. Reassortant EU H1av viruses containing the M gene from the H1pdm09 lineage were also detected in Belgium during this study period.

The predominance of the EU H1av viruses and low incidences of the H1pdm09 viruses have also been reported in countries across mainland Europe^21,22,41–44^. The EU H1av swIAV were found in proportions ranging between 27.9% and 69.6% in these countries, while the H1pdm09 swIAV accounted for up to 9.1% of the circulating strains. In the United Kingdom and the Republic of Ireland, in contrast, the H1pdm09 viruses circulate at high frequencies of 36% and 56.3% respectively^22^.

Full-length genomes of 18 isolates revealed the presence of previously reported genotypes^21,22,43^, with all except one virus containing the M gene from the H1pdm09 lineage. swIAV circulating in European pigs continue to exchange genes amongst each other resulting in second generation reassortants with different genotypes from their precursors. Especially, reassortants with the H1pdm09 viruses being on the rise, as these have also been reported in other studies. The three new genotypes of swIAV that were identified in this study resulted from the replacement of the M gene of the EU H1av viruses with that of the H1pdm09 virus. swIAV genotypes with the M gene from the H1pdm09 lineage have become established in swIAV throughout Europe. In fact, in the study by Henritzi and colleagues^22^, EU H1av viruses carrying the M gene from the H1pdm09 had the second-highest prevalence of genome constellations. Experimental studies in guinea pig and ferret models have associated the M gene from the H1pdm09 viruses with efficient transmission of influenza viruses^45–47^. Using reverse genetics to introduce the M gene of the H1pdm09 virus A/Netherlands/602/2009 into a background of A/Puerto Rico/8/1934 (H1N1), Campbell and colleagues found an increase in NA activity. This H1pdm09 M-NA interaction is thought to lead to efficient transmission of influenza A viruses^45^. In the United States, H3N2 variant (H3N2v) viruses that contained the M gene from the H1pdm09 virus caused human infections in 2011–2012^48^ and in 2016^49,50^. Although influenza variant viruses rarely result in sustained human-to-human transmissions, this raises concerns about the pandemic potential of these variant viruses. The role of the M gene from the H1pdm09 virus in H1 swIAV therefore, needs to be explored further.

The antigenic evolution within both the EU H1av and the EU H1hu swIAV was observed to be slow, in that the antisera against the past reference viruses isolated as early as 1982 and 1986 still reacted with the viruses isolated in 2014–2019. Despite this, the rate of evolution of the EU H1av was slightly faster than that of the EU H1hu swIAV isolates, both at the antigenic level (0.15 AU per year vs 0.10 AU per year) and at the genetic level (4.06 × 10^–3^ substitutions per site per year vs 3.76 × 10^–3^ substitutions per site per year aa substitutions). Amino acid substitution E155G has been previously shown to affect receptor binding specificity of H1 influenza viruses^38,51,52^. Most of the EU H1av isolates (10 out of 13) with this aa substitution had great antigenic distances (5.0–6.8 AU) from their ancestor virus. While this may not be the sole factor influencing the observed antigenic changes, we speculate that the role of this substitution in receptor binding specificity impacted HI results and hence the increased antigenic distances. The EU H1hu isolates and their human ancestor virus, on the other hand, shared the same aa among the ones considered important. Yet great antigenic distances were observed between the EU H1hu isolates and the US H1δ swIAV as well as the recent human viruses (NC99 and BR07). Interestingly, aa substitution F71I was observed between these viruses and the EU H1hu isolates. Amino acid substitution at position 71 has been shown to drive antigenic drift both in swine and human H1 influenza viruses^38,39^, and could explain the antigenic distances observed. Three out of the five H1pdm09 isolates were antigenically divergent from the H1pdm09 reference viruses (4.8 – 12.9 AU). These three isolates shared aa substitutions at position 187 (D187S or D187N). Substitution D187N has been shown to correlate with antigenic changes^38^. Position 187 is also part of the receptor-binding site^53^ meaning that these substitutions could influence receptor-binding capacity of these viruses and lead to changes in HI titers. This could possibly explain the observed antigenic divergence of the three isolates. An additional aa substitution was N156D, which was present in the isolate swG235/19, and has been associated with large changes in biophysical properties of the H1pdm09 viruses^40^. This substitution could be the reason why this isolate was antigenically different from the rest. Although the described amino acid substitutions, which are mainly from previous studies may have been the cause of the observed antigenic differences in this study, we cannot rule out the fact that other mutations that we observed but have not been previously described, also impacted the antigenicity of the isolates.

As explained earlier, introduction of swine viruses to humans poses a risk to public health. We recently reported a case of human infection with one of the EU H1av isolates from the current study, A/swine/Netherlands/Gent-193/2019 (Fig. 1) in a farmer in the Netherlands^54^. What draws our attention is the lack of antibodies against these viruses in the human population. A recent serological study showed that humans largely lack HI and virus neutralizing antibodies against the EU H1av swIAV, with seroprevalence rates against these viruses being ≤ 10%^55^. Henritzi and colleagues, in contrast, reported low neutralizing activity of human plasma against only a few strains of H1 avian-like viruses, while the majority of adult human sera revealed moderate to high titers^22^. If these viruses would acquire the ability to replicate and transmit in humans, they would have considerable pandemic potential. Apart from the EU H1av viruses, the EU H1hu viruses may also pose a threat to humans. The antigenic clustering of the EU H1hu isolates with the oldest human H1 virus from 1986 and not with the more recent human H1 viruses isolated in 1999 or later, indicate a slow evolution of these human-derived viruses in swine. Swine, therefore, serve as reservoirs for the past viruses that ceased to circulate in humans. Over the years, the human population immunity against these swIAV will gradually decrease. Because the younger human population will be lacking immunity against the circulating swIAV that were initially introduced from humans, there will be an increased chance of re-introduction of these viruses into humans. Although re-introduction of a virus to a population that lacks immunity rarely results in a pandemic, such a possibility cannot be ignored. This was illustrated by the 2009 pandemic virus. This virus inherited its HA from a North American swIAV, which is a descendant of the 1918 pandemic H1N1 virus. In 2009, only humans born before 1930–40 had cross-reactive antibodies against the new pandemic virus, so the virus could easily spread among the younger population^56^. Such a scenario could occur in the future with other swIAV, if these viruses will manage to adapt to, replicate and become transmissible in humans. The results of this study stress the importance of frequent surveillance of swIAV, as well as monitoring of the evolution of these viruses for better preparedness in the event of a pandemic.

## Supplementary Information

Supplementary Information 1.

Supplementary Information 2.

Supplementary Information 3.

Supplementary Information 4.

Supplementary Information 5.

Supplementary Information 6.

## Data Availability

Nucleic acid sequences generated during this study are available in the GenBank repository and have been assigned accession numbers MW362568-MW362741. . Data generated or analyzed during this study are included in this article and as supplementary information.
