# Supplementary material for: Genetic and antigenic evolution of H1 swine influenza A viruses isolated in Belgium and the Netherlands from 2014 through 2019
Source: Sci Rep. 2021 May 28;11:11276. doi: 10.1038/s41598-021-90512-z (PMC8163766; doi:10.1038/s41598-021-90512-z)
Supplement: Supplementary file 1 — Supplementary Information 1. [file 41598_2021_90512_MOESM1_ESM.pdf]

Genetic and antigenic evolution of H1 swine influenza A viruses isolated in Belgium and the Netherlands from 2014 through 2019

Sharon Chepkwony, Anna Parys, Elien Vandoorn, Wojciech Stadejek, Jiexiong Xie, Jacqueline King, Annika Graaf, Anne Pohlmann, Martin Beer, Timm Harder and Kristien Van Reeth

Supplementary Table S1. Range of percentage amino acid (aa) identities in the HA1 and antigenic sites of the isolates and reference viruses

**A** Virus Isolates clade	EU H1av reference viruses					
	swFI82 (Clade 1C.1)		swBE98 (Clade 1C.2)		swG05, swG10 (Clade 1C.2.1)	
	HA1	Antigenic sites	HA1	Antigenic sites	HA1	Antigenic sites
1C.2.1	85.3 – 88.7	70.0 – 82.0	91.1 – 94.6	78.0 – 90.0	92.0 – 96.8	82.0 – 96.0
1C.2.2	84.1 – 87.2	76.0 – 82.0	88.7 – 91.7	84.0 – 90.0	91.1 – 95.1	88.0 – 96.0
1B.1.2.1	71.9 – 73.7	34.0 – 40.0	69.4 – 70.9	40.0 – 44.0	68.5 – 70.6	36.0 – 42.0
1A.3.3.2	74.0 – 77.7	52.0 – 66.0	70.9 – 74.0	58.0 – 68.0	69.7 – 73.4	54.0 – 64.0

**B** Virus isolates clade	EU H1hu and related human seasonal reference viruses					
	swSC94 (Clade 1B.1)		swG99, swG02, swG12 (Clade 1B.1.2.1)		TW86 (Clade 1B.1-like)	
	HA1	Antigenic sites	HA1	Antigenic sites	HA1	Antigenic sites
1C.2.1	69.7 – 72.4	40.0 – 48.0	68.2 – 71.2	34.0 – 44.0	70.6 – 73.6	40.0 – 48.0
1C.2.2	69.1 – 70.9	42.0 – 44.0	67.3 – 70.0	36.0 – 40.0	70.6 – 72.5	40.0 – 44.0
1B.1.2.1	89.2 – 91.1	72.0 – 86.0	87.5 – 96.0	74.0 – 94.0	89.0 – 90.5	76.0 – 82.0
1A.3.3.2	70.0 – 72.2	36.0 – 46.0	68.5 – 71.9	32.0 – 42.0	70.3 – 73.1	36.0 – 44.0

**C** Virus isolates clade	US H1δ and related human seasonal reference viruses					
	swIL10 (Clade 1B.2.2.2)		swAL16 (Clade 1B.2.2.1)		NC99, BR07 (Other human 1B.2)	
	HA1	Antigenic sites	HA1	Antigenic sites	HA1	Antigenic sites
1C.2.1	70.2 – 72.9	42.0 – 48.0	69.6 – 72.4	46.0 – 52.0	69.6 – 73.6	40.0 – 48.0
1C.2.2	69.0 – 70.9	40.0 – 44.0	69.0 – 70.9	44.0 – 48.0	69.3 – 72.1	40.0 – 44.0
1B.1.2.1	82.5 – 84.6	58.0 – 70.0	80.4 – 82.5	60.0 – 68.0	82.8 – 84.9	58.0 – 70.0
1A.3.3.2	70.6 – 72.4	42.0 – 50.0	70.9 – 73.0	42.0 – 50.0	70.6 – 72.7	38.0 – 48.0

**D** Virus isolates clade	H1pdm09 and US H1γ reference viruses			
	CA09, SL15 (Clade 1A.3.3.2)		swOH07 (Clade 1A.3.3.3)	
	HA1	Antigenic sites	HA1	Antigenic sites
1C.2.1	68.8 – 73.7	56.0 – 72.0	71.9 – 74.6	60.0 – 70.0
1C.2.2	71.6 – 74.3	60.0 – 68.0	73.4 – 74.3	64.0 – 66.0
1B.1.2.1	68.8 – 71.6	32.0 – 44.0	70.3 – 71.6	36.0 – 42.0
1A.3.3.2	88.7 – 97.2	68.0 – 94.0	84.4 – 89.3	66.0 – 80.0

The reference viruses include the European avian-like H1 (EU H1av) (**A**), the European human-like H1 (EU H1hu) and related human H1 (other human 1B.1-like) (**B**), the United States H1δ (US H1δ) and related human H1 (other human 1B.2) (**C**) and the 2009 pandemic H1 (H1pdm09) and US H1γ viruses (**D**). Full virus names: swFI82 -

A/swine/Finestère/2899/1982; swBE98 - A/swine/Belgium/1/1998; swG05 - A/swine/Gent/132/2005; swG10 - A/swine/Gent/28/2010; swSC94 - A/swine/Scotland/410440/1994; swG99 - A/swine/Gent/7625/1999; G02 - A/swine/Gent/177/2002; G12 - A/swine/Gent/26/2012; TW86 - A/Taiwan/1/1986; swIL10 - A/swine/Illinois/A0104020/2010; swAL16 - A/swine/Alabama/A01104091/2016; NC99 - A/New Caledonia/20/1999; BR07 - A/Brisbane/59/2007; CA09 - A/California/04/2009; SL15 - A/Slovenia/2903/2015; OH07 - A/swine/Ohio/511445/2007
