# Supplementary material for: Genetic and antigenic evolution of H1 swine influenza A viruses isolated in Belgium and the Netherlands from 2014 through 2019
Source: Sci Rep. 2021 May 28;11:11276. doi: 10.1038/s41598-021-90512-z (PMC8163766; doi:10.1038/s41598-021-90512-z)
Supplement: Supplementary file 2 — Supplementary Information 2. [file 41598_2021_90512_MOESM2_ESM.pdf]

Genetic and antigenic evolution of H1 swine influenza A viruses isolated in Belgium and the Netherlands from 2014 through 2019

Sharon Chepkwony, Anna Parys, Elien Vandoorn, Wojciech Stadejek, Jiexiong Xie, Jacqueline King, Annika Graaf, Anne Pohlmann, Martin Beer, Timm Harder and Kristien Van Reeth

Supplementary Table S2. Antigenic relatedness of H1 swIAV isolated in 2014-2019 in Belgium and the Netherlands to European and North American swIAV and to human influenza viruses.

			HI titer with hyperimmune swine or ferret* serum against:															
			Eu H1av				Eu H1hu				H1pdm09		US γ/δ H1			Human H1		
		Lineage	Sw FI82	sw BE98	sw G05	Sw G10	sw SC94	Sw G99	Sw G02	sw G12	CA09	SL15	sw OH07	sw IL10	Sw AL16	TW86*	NC99*	BR07*
Reference viruses	A/swine/Finestère/2899/1982 (swFI82)	Eu H1av	**10240**	160	160	40	<10	40	<10	<10	160	640	40	<10	<10	<10	<10	<10
	A/swine/Belgium/1/1998 (swBE98)	Eu H1av	10240	**5120**	1280	80	<10	160	<10	<10	80	10	10	<10	<10	<10	<10	<10
	A/swine/Gent/132/2005 (swG05)	Eu H1av	160	80	**320**	20	<10	10	<10	<10	10	320	<10	<10	<10	<10	<10	<10
	A/swine/Gent/28/2010 G10	Eu H1av	320	160	320	**640**	<10	<10	<10	<10	40	5120	80	<10	<10	<10	<10	<10
	A/swine/Scotland/410440/1994 (swSC94)	Eu H1hu	20	10	20	<10	**160**	2560	1280	1280	160	80	20	20	40	160	40	<10
	A/swine/Gent/7625/1999 (swG99)	Eu H1hu	10	10	20	<10	40	**5120**	2560	1280	20	<10	<10	<10	<10	160	10	<10
	A/swine/Gent/177/2002 (swG02)	Eu H1hu	10	10	20	10	160	5120	**10240**	2560	80	<10	<10	<10	<10	160	20	<10
	A/swine/Gent/26/2012 (swG12)	Eu H1hu	10	<10	20	<10	80	2560	5120	**5120**	40	<10	<10	<10	<10	640	20	<10
	A/California/04/2009 (CA09)	H1pdm09	640	80	80	40	<10	80	<10	<10	**2560**	10240	1280	<10	40	<10	<10	<10
	A/Slovenia/2903/2015 (SL15)	H1pdm09	640	40	40	40	<10	160	<10	<10	5120	**10240**	1280	<10	10	<10	<10	<10
	A/swine/Ohio/511445/2007 (swOH07)	US γ H1	640	40	80	20	<10	80	<10	<10	160	2560	**1280**	<10	<10	<10	<10	<10
	A/swine/Illinois/A01047020/2010 (swIL10)	US δ1b H1	<10	<10	<10	<10	<10	<10	<10	<10	<10	<10	<10	**1280**	10	<10	80	640
	A/swine/Alabama/A01104091/2016 (swAL16)	US δ1a H1	<10	<10	<10	<10	<10	40	80	20	10	<10	<10	160	**640**	<10	640	40
	A/Taiwan/1/1986 (TW86)	Human H1	10	10	<10	<10	80	640	1280	640	40	<10	<10	<10	<10	**1280**	20	<10
	A/New Caledonia/20/1999 (NC99)	Human H1	<10	<10	<10	<10	<10	40	<10	40	<10	<10	<10	40	10	<10	**640**	160
	A/Brisbane/59/2007 (BR07)	Human H1	<10	<10	<10	<10	<10	10	<10	<10	<10	<10	<10	80	20	<10	80	**2560**
Virus isolate	A/swine/Netherlands/Gent-5/2014 (swG5/14)	Eu H1av	160	320	160	80	<10	<10	<10	<10	20	<10	<10	<10	<10	<10	<10	<10
	A/swine/Gent/16/2014 (swG16/14)	Eu H1av	160	640	160	320	<10	<10	<10	<10	20	<10	<10	<10	<10	<10	<10	<10

		HI titer with hyperimmune swine or ferret* serum against:															
		Eu H1av				Eu H1hu				H1pdm09		US γ/δ H1			Human H1		
	Lineage	Sw Fl82	sw BE98	sw G05	Sw G10	sw SC94	Sw G99	Sw G02	sw G12	CA09	SL15	sw OH07	sw IL10	Sw AL16	TW86*	NC99*	BR07*
A/swine/Gent/36/2014 (swG36/14)	Eu H1av	320	320	320	40	<10	<10	<10	<10	20	<10	20	<10	<10	<10	<10	<10
A/swine/Netherlands/Gent-46/2014 (swG46/14)	Eu H1av	320	640	320	80	<10	<10	<10	<10	20	<10	<10	<10	<10	<10	<10	<10
A/swine/Gent/55/2014 (swG55/14)	Eu H1av	320	640	320	40	<10	<10	<10	<10	1280	160	<10	<10	<10	<10	<10	<10
A/swine/Gent/61/2014 (swG61/14)	Eu H1av	320	640	320	40	<10	<10	<10	<10	640	80	<10	<10	<10	<10	<10	<10
A/swine/Gent/78/2014 (swG78/14)	Eu H1av	320	640	320	80	<10	<10	<10	<10	80	20	20	<10	<10	<10	<10	<10
A/swine/Gent/121/2014 (swG121/14)	Eu H1av	320	640	640	80	<10	<10	<10	<10	40	<10	<10	<10	<10	<10	<10	<10
A/swine/Gent/122/2014 (swG122/14)	Eu H1av	320	640	640	80	<10	<10	<10	<10	40	<10	<10	<10	<10	<10	<10	<10
A/swine/Gent/48/2015 (swG48/15)	Eu H1av	320	320	320	80	<10	<10	<10	<10	20	<10	<10	<10	<10	<10	<10	<10
A/swine/Netherlands/Gent-62/2015 (swG62/15)	Eu H1av	160	640	640	80	<10	<10	<10	<10	10	<10	<10	<10	<10	<10	<10	<10
A/swine/Gent/173/2015 (swG173/15)	Eu H1av	160	1280	320	80	<10	<10	<10	<10	20	<10	<10	<10	<10	<10	<10	<10
A/swine/Gent/150/2016 (swG150/16)	Eu H1av	640	160	640	80	<10	10	<10	<10	20	<10	<10	<10	<10	<10	<10	<10
A/swine/Gent/13/2017 (swG13/17)	Eu H1av	1280	1280	1280	160	<10	<10	<10	<10	40	1280	160	<10	<10	<10	<10	<10
A/swine/Gent/121/2017 (swG121/17)	Eu H1av	640	320	320	40	<10	40	<10	<10	1280	40	20	<10	20	<10	<10	<10
A/swine/Gent/138/2017 (swG138/17)	Eu H1av	1280	640	640	80	<10	20	<10	<10	80	<10	<10	10	<10	<10	<10	<10
A/swine/Netherlands/Gent-157/2017 (swG157/17)	Eu H1av	1280	160	640	160	<10	<10	<10	<10	40	1280	80	<10	<10	<10	<10	<10
A/swine/Gent/180/2017 (G180/17)	Eu H1av	1280	640	160	80	<10	40	<10	<10	640	20	<10	<10	<10	<10	<10	<10
A/swine/Netherlands/Gent-8/2018 (swG8/18)	Eu H1av	1280	640	1280	80	<10	<10	<10	<10	40	2560	160	<10	<10	<10	<10	<10
A/swine/Netherlands/Gent-9-p20/2018 (swG9-20/18)	Eu H1av	640	320	320	40	<10	40	<10	<10	1280	20	<10	<10	<10	<10	<10	<10
A/swine/Netherlands/Gent-31/2018 (swG31/18)	Eu H1av	640	640	640	160	<10	<10	<10	<10	20	1280	80	<10	<10	<10	<10	<10
A/swine/Netherlands/Gent-32/2018 (G32/18)	Eu H1av	1280	160	1280	160	<10	20	<10	<10	40	5120	160	<10	<10	<10	<10	<10
A/swine/Netherlands/Gent-103/2018 (swG103/18)	Eu H1av	640	320	640	80	<10	<10	<10	<10	40	1280	40	<10	<10	<10	<10	<10
A/swine/Gent/127/2018 (swG127/18)	Eu H1av	640	640	640	80	<10	<10	<10	<10	40	320	20	<10	<10	<10	<10	<10
A/swine/Gent/196/2018 (swG196/18)	Eu H1av	80	320	160	40	<10	<10	<10	<10	20	<10	10	<10	<10	<10	<10	<10
A/swine/Gent/241/2018 (swG241/18)	Eu H1av	320	640	320	40	<10	<10	<10	<10	<10	<10	<10	<10	<10	<10	<10	<10
A/swine/Gent/243/2018 (swG243/18)	Eu H1av	640	320	640	80	<10	<10	<10	<10	40	640	<10	<10	<10	<10	<10	<10

	Lineage	HI titer with hyperimmune swine or ferret* serum against:															
		Eu H1av				Eu H1hu				H1pdm09		US γ/δ H1			Human H1		
		Sw Fl82	sw BE98	sw G05	Sw G10	sw SC94	Sw G99	Sw G02	sw G12	CA09	SL15	sw OH07	sw IL10	Sw AL16	TW86*	NC99*	BR07*
A/swine/Gent/5/2019 (swG5/19)	Eu H1av	640	640	320	40	<10	20	<10	<10	20	<10	<10	<10	<10	<10	<10	<10
A/swine/Gent/6/2019 (swG6/19)	Eu H1av	320	640	320	40	<10	40	<10	<10	<10	<10	<10	<10	<10	<10	<10	<10
A/swine/Gent/9/2019 (swG9/19)	Eu H1av	320	640	640	80	<10	10	<10	<10	80	1280	<10	<10	<10	<10	<10	<10
A/swine/Gent/29/2019 (swG29/19)	Eu H1av	320	640	640	160	<10	20	<10	<10	20	<10	40	<10	<10	<10	<10	<10
A/swine/Gent/31/2019 (swG31/19)	Eu H1av	320	320	640	80	<10	<10	<10	<10	40	<10	10	<10	<10	<10	<10	<10
A/swine/Gent/54/2019 (swG54/19)	Eu H1av	160	320	320	40	<10	<10	<10	<10	40	<10	<10	<10	<10	<10	<10	<10
A/swine/Gent/99/2019 (swG99/19)	Eu H1av	320	640	640	80	<10	<10	<10	<10	40	10	<10	<10	<10	<10	<10	<10
A/swine/Gent/124/2019 (swG124/19)	Eu H1av	160	160	640	40	<10	<10	<10	<10	40	<10	<10	<10	<10	<10	<10	<10
A/swine/Gent/184/2019 (swG184/19)	Eu H1av	80	320	320	80	<10	<10	<10	<10	20	<10	10	<10	<10	<10	<10	<10
A/swine/Netherlands/Gent-193/19 (swG193/19)	Eu H1av	40	640	640	80	<10	40	<10	<10	1280	320	10	<10	<10	<10	<10	<10
A/swine/Gent/202/19 (swG202/19)	Eu H1av	40	640	320	80	<10	10	<10	<10	20	<10	20	<10	<10	<10	<10	<10
A/swine/Gent/203/19 (swG203/19)	Eu H1av	80	1280	640	160	<10	20	<10	<10	80	20	20	<10	<10	<10	<10	<10
A/swine/Gent/204/19 (swG204/19)	Eu H1av	40	640	640	80	<10	10	<10	<10	20	<10	20	<10	<10	<10	<10	<10
A/swine/Gent/205/19 (swG20519)	Eu H1av	20	640	320	40	<10	10	<10	<10	20	<10	<10	<10	<10	<10	<10	<10
A/swine/Gent/208/19 (swG208/19)	Eu H1av	40	640	320	40	<10	<10	<10	<10	10	<10	<10	<10	<10	<10	<10	<10
A/swine/Gent/236/19 (swG236/19)	Eu H1av	410	320	320	80	<10	<10	<10	<10	10	<10	<10	<10	<10	<10	<10	<10
A/swine/Gent/228/2014 (swG228/14)	H1pdm09	20	<10	<10	<10	<10	20	<10	<10	160	80	<10	10	<10	<10	<10	<10
A/swine/Gent/121/2018 (swG121/18)	H1pdm09	640	80	80	20	10	10	<10	<10	1280	5120	80	<10	<10	<10	<10	<10
A/swine/Gent/53/2019 (swG53/19)	H1pdm09	320	40	40	20	<10	20	<10	<10	1280	1280	20	<10	<10	<10	<10	<10
A/swine/Gent/220/19 (swG220/19)	H1pdm09	<10	<10	20	<10	<10	<10	<10	<10	1280	320	<10	<10	<10	<10	<10	<10
A/swine/Gent/235/19 (swG235/19)	H1pdm09	<10	<10	<10	<10	<10	10	<10	<10	<10	<10	<10	<10	<10	<10	<10	<10
A/swine/Gent/30/2014 (swG30/14)	Eu H1hu	<10	<10	<10	<10	320	2560	2560	640	10	<10	<10	<10	<10	40	10	<10
A/swine/Gent/35/2014 (swG35/14)	Eu H1hu	<10	<10	<10	<10	160	2560	1280	640	10	<10	<10	<10	<10	40	10	<10
A/swine/Gent/93/2014 (swG93/14)	Eu H1hu	<10	20	<10	<10	640	5120	5120	2560	40	<10	<10	<10	<10	40	10	<10

		HI titer with hyperimmune swine or ferret* serum against:															
		Eu H1av				Eu H1hu				H1pdm09		US γ/δ H1			Human H1		
	Lineage	Sw Fl82	sw BE98	sw G05	Sw G10	sw SC94	Sw G99	Sw G02	sw G12	CA09	SL15	sw OH07	sw IL10	Sw AL16	TW86*	NC99*	BR07*
A/swine/Gent/94/2014 (swG94/14)	Eu H1hu	20	<10	<10	<10	80	2560	2560	1280	40	10	<10	80	<10	40	10	<10
A/swine/Gent/175/2014 (swG175/14)	Eu H1hu	<10	10	<10	<10	320	5120	5120	1280	20	<10	<10	<10	<10	80	20	<10
A/swine/Gent/36/2016 [a] (swG36/16)	Eu H1hu	10	10	<10	<10	80	10240	2560	5120	80	10	<10	<10	<10	320	40	<10
A/swine/Gent/5/2017 [a] (swG5/17)	Eu H1hu	10	10	<10	<10	160	5120	5120	5120	80	<10	<10	<10	<10	640	80	<10
A/swine/Netherlands/Gent-7/2018 (swG7/18)	Eu H1hu	<10	<10	<10	<10	<10	640	80	160	<10	<10	<10	<10	<10	10	<10	<10
A/swine/Netherlands/Gent-9p18/2018 (swG9-18/18)	Eu H1hu	<10	20	<10	<10	20	2560	1280	640	<10	<10	<10	<10	10	20	80	<10
A/swine/Gent/77/2018 (swG77/18)	Eu H1hu	10	40	<10	<10	2560	5120	2560	640	<10	<10	<10	<10	<10	160	20	<10
A/swine/Gent/180/2018 (swG180/18)	Eu H1hu	10	20	<10	<10	1280	5120	2560	1280	40	<10	<10	<10	<10	160	20	<10
A/swine/Netherlands/Gent-185/2018 (swG185/18)	Eu H1hu	10	10	<10	<10	640	2560	1280	640	10	<10	<10	<10	<10	40	10	<10
A/swine/Gent/189/2018 (swG189/18)	Eu H1hu	<10	10	<10	<10	20	320	40	40	<10	<10	<10	<10	<10	<10	<10	<10
A/swine/Gent/216/2018 (swG216/18)	Eu H1hu	<10	20	<10	<10	80	1280	320	320	<10	<10	<10	10	<10	<10	40	<10
A/swine/Gent/233/2018 (swG233/18)	Eu H1hu	<10	<10	<10	<10	40	640	640	320	40	10	<10	40	<10	10	10	<10
A/swine/Gent/28/2019 (swG28/19)	Eu H1hu	20	<10	<10	<10	160	2560	1280	640	80	20	<10	80	<10	80	20	<10
A/swine/Gent/56/2019 (swG56/19)	Eu H1hu	<10	20	<10	<10	320	2560	2560	1280	40	<10	<10	<10	<10	80	40	<10
A/swine/Gent/76/2019 (swG75/19)	Eu H1hu	<10	10	10	<10	80	2560	2560	1280	10	<10	<10	<10	<10	20	10	<10
A/swine/Gent/114/2019 (swG114/19)	Eu H1hu	<10	20	<10	<10	320	2560	2560	1280	10	<10	<10	<10	<10	320	20	<10
A/swine/Gent/119/2019 (swG119/19)	Eu H1hu	<10	20	<10	<10	320	5120	5120	2560	40	<10	<10	<10	<10	160	40	<10
A/swine/Gent/178/2019 (swG178/19)	Eu H1hu	<10	20	<10	<10	320	5120	2560	1280	40	<10	<10	<10	<10	160	40	<10
A/swine/Gent/180/2019 (swG180/19)	Eu H1hu	<10	20	<10	<10	320	5120	5120	1280	40	<10	<10	<10	<10	160	40	<10
A/swine/Gent/206/19 (swG206/19)	Eu H1hu	<10	<10	<10	<10	80	5120	2560	640	320	10	<10	160	<10	20	10	<10
