# Supplementary material for: Genetic and antigenic evolution of H1 swine influenza A viruses isolated in Belgium and the Netherlands from 2014 through 2019
Source: Sci Rep. 2021 May 28;11:11276. doi: 10.1038/s41598-021-90512-z (PMC8163766; doi:10.1038/s41598-021-90512-z)
Supplement: Supplementary file 3 — Supplementary Information 3. [file 41598_2021_90512_MOESM3_ESM.pdf]

Genetic and antigenic evolution of H1 swine influenza A viruses isolated in Belgium and the Netherlands from 2014 through 2019

Sharon Chepkwony, Anna Parys, Elien Vandoorn, Wojciech Stadejek, Jiexiong Xie, Jacqueline King, Annika Graaf, Anne Pohlmann, Martin Beer, Timm Harder and Kristien Van Reeth

Supplementary Table S3. Antigenic distances between the H1 isolates and the reference viruses

		**Reference viruses**															
		**EU H1av**				**EU H1hu**				**H1pdm09**		**US H1γ**	**US H1δ**		**Human H1**		
		**swFI82**	**swBE98**	**swG05**	**swG10**	**swSC94**	**swG99**	**swG02**	**swG12**	**CA09**	**SL15**	**swOH07**	**swIL10**	**swAL16**	**TW86**	**NC99**	**BR07**
Reference viruses	A/swine/Finistere/2899/1982_H1N1_Clade_1C.1HA																
	A/swine/Belgium/1/1998_H1N1_Clade_1C.2HA	3.9															
	A/swine/Gent/132/2005_H1N1_Clade_1C.2.1HA	4.5	5.2														
	A/swine/Gent/28/2010_H1N1_Clade_1C.2.1HA	1.7	4.5	3.1													
	A/swine/Scotland/410440/1994_H1N2_Clade_1B.1HA	8.2	7.7	10.2	8.5												
	A/swine/Gent/7625/1999_H1N2_Clade_1B.1.2.1HA	9.6	8.2	11.6	10.1	2.5											
	A/swine/Gent/177/2002_H1N2_Clade_1B.1.2.1HA	9.3	8.2	11.5	9.7	1.9	0.7										
	A/swine/Gent/26/2012_H1N2_Clade_1B.1.2.1HA	9.9	9.2	12.2	10.3	2.1	2.0	1.4									
	A/California/04/2009_H1N1_Clade_1A.3.3.2HA	3.3	5.7	5.6	3.0	6.2	8.2	7.8	8.0								
	A/Slovenia/2903/2015_H1N1_Clade_1A.3.3.2HA	3.5	6.0	5.9	3.3	6.3	8.3	7.8	8.0	0.4							
	A/swine/Ohio/511445/2007_H1N1_Clade_1A.3.3.3HA	2.8	5.9	6.5	3.4	7.1	8.9	8.4	8.6	2.1	1.9						
	A/swine/Illinois/A01047020/2010_H1N2_Clade_1B.2.2.2HA	14.2	13.3	13.5	13.4	8.6	9.3	9.4	9.5	11.7	11.9	13.6					
	A/swine/Alabama/A01104091/2016_H1N2_Clade_1B.2.2.1HA	11.2	9.6	10.8	10.7	5.8	6.2	6.4	6.9	9.3	9.5	11.0	4.0				
	A/Taiwan/1/1986_H1N1_Clade_Other_human_1B.2HA	9.9	9.9	12.6	10.4	2.9	3.6	3.0	1.7	7.9	7.8	8.2	10.3	8.2			
	A/New_Caledonia/20/1999_H1N1_Clade_Other_human_1B.2HA	13.6	11.6	13.2	13.2	7.5	7.2	7.5	8.1	11.8	12.0	13.4	4.3	2.7	9.5		
	A/Brisbane/59/2007_H1N1_Clade_Other_human_1B.2HA	14.8	13.4	13.9	14.0	9.1	9.4	9.6	9.8	12.6	12.8	14.4	1.8	3.8	10.9	3.1	
Virus isolates	A/swine/Netherlands/Gent_05/2014_H1N1_Clade_1C.2.1HA	6.3	3.4	4.8	6.1	9.7	10.1	10.2	11.3	7.8	8.2	8.6	13.1	9.6	12.3	11.4	13.0
	A/swine/Gent/16/2014_H1N1_Clade_1C.2.1HA	5.8	2.6	4.9	5.8	9.4	9.7	9.8	11.0	7.5	7.9	8.1	13.4	9.8	11.9	11.6	13.3
	A/swine/Gent/36/2014_H1N1_Clade_1C.2.1HA	5.5	2.8	4.2	5.4	9.8	10.3	10.3	11.4	7.3	7.7	7.9	13.7	10.2	12.3	12.1	13.6
	A/swine/Netherlands/Gent_46/2014_H1N1_Clade_1C.2.1HA	5.7	2.7	4.7	5.7	9.6	10.0	10.1	11.2	7.5	7.9	8.1	13.6	10.1	12.1	11.8	13.5
	A/swine/Netherlands/Gent_55/2014_H1N1_Clade_1C.2.1HA	4.3	4.5	2.5	3.0	7.9	9.4	9.2	10.0	4.1	4.5	5.6	11.2	8.5	10.4	10.9	11.7
	A/swine/Gent/61/2014_H1N1_Clade_1C.2.1HA	4.5	4.3	2.3	3.3	8.2	9.6	9.4	10.2	4.7	5.1	6.1	11.3	8.5	10.8	10.9	11.7
	A/swine/Gent/78/2014_H1N1_Clade_1C.2.1HA	4.6	3.1	2.7	4.0	9.0	9.9	9.9	10.9	5.9	6.3	6.9	12.7	9.5	11.6	11.6	12.8
	A/swine/Gent/121/2014_H1N1_Clade_1C.2.1HA	5.5	2.7	4.2	5.3	9.2	9.7	9.8	10.9	7.0	7.4	7.8	13.0	9.5	11.8	11.4	12.9
	A/swine/Gent/122/2014_H1N1_Clade_1C.2.1HA	5.5	2.7	4.2	5.3	9.2	9.7	9.8	10.9	7.0	7.4	7.8	13.0	9.5	11.8	11.4	12.9
	A/swine/Gent/48/2015_H1N1_Clade_1C.2.1HA	5.8	3.0	4.4	5.6	9.7	10.2	10.3	11.4	7.5	7.9	8.2	13.4	10.0	12.3	11.8	13.4
	A/swine/Netherlands/Gent_62/2015_H1N1_Clade_1C.2.1HA	5.9	2.8	4.7	5.8	9.6	10.0	10.1	11.2	7.6	8.0	8.2	13.5	9.9	12.1	11.7	13.4
	A/swine/Gent/173/2015_H1N1_Clade_1C.2.1HA	5.9	2.6	5.0	5.9	9.3	9.6	9.7	10.9	7.5	7.9	8.1	13.4	9.8	11.8	11.5	13.2

		Reference viruses															
		**EU H1av**				**EU H1hu**				**H1pdm09**		**US H1γ**	**US H1δ**		**Human H1**		
		**swFI82**	**swBE98**	**swG05**	**swG10**	**swSC94**	**swG99**	**swG02**	**swG12**	**CA09**	**SL15**	**swOH07**	**swIL10**	**swAL16**	**TW86**	**NC99**	**BR07**
	A/swine/Gent/150/2016_H1N1_Clade_1C.2.1HA	5.6	2.9	4.1	5.3	9.4	9.9	10.0	11.1	7.1	7.5	7.9	13.1	9.6	12.0	11.5	13.0
	A/swine/Gent/13/2017_H1N1_Clade1C.2.1HA	1.5	3.0	3.3	1.5	8.2	9.5	9.2	10.0	3.7	4.0	3.9	13.5	10.4	10.2	12.7	13.9
	A/swine/Gent/121/2017_H1avN2_Clade1C.2.1HA	4.6	4.2	3.9	3.7	6.6	8.0	7.9	8.6	3.9	4.3	5.5	10.2	7.2	9.2	9.7	10.6
	A/swine/Gent/138/2017_H1N1_Clade1C.2.1HA	5.5	2.8	4.6	5.1	7.5	8.2	8.2	9.3	6.1	6.5	7.2	11.3	7.8	10.2	9.7	11.3
	A/swine/Gent/180/2017_H1N1_Clade1C.2.1HA	4.9	3.3	3.9	4.3	7.1	8.1	8.1	9.1	5.0	5.5	6.4	10.7	7.5	9.8	9.7	10.9
	A/swine/Netherlands/Gent_8/2018_H1N1_Clade1C.2.1HA	1.2	3.8	3.4	0.8	8.3	9.8	9.5	10.1	3.3	3.6	3.4	13.7	10.7	10.3	13.1	14.2
	A/swine/Netherlands/Gent_9-p20/2018_H1N1_Clade_1C.2.1HA	5.0	3.9	3.7	4.1	7.1	8.3	8.2	9.1	4.7	5.1	6.2	10.4	7.3	9.8	9.6	10.6
	A/swine/Gent/127/2018_H1N1_Clade_1C.2.1HA	2.9	3.4	1.9	2.2	9.1	10.4	10.2	11.0	4.7	5.1	5.3	13.5	10.5	11.4	12.8	13.9
	A/swine/Gent/196/2018_H1N1_Clade_1C.2.1HA	6.5	3.6	4.8	6.2	9.4	9.8	10.0	11.1	7.7	8.1	8.7	12.6	9.1	12.1	10.9	12.4
	A/swine/Gent/241/2018_H1N1_Clade_1C.2.1HA	6.0	2.8	5.4	6.2	10.2	10.4	10.5	11.7	8.1	8.5	8.4	14.6	11.0	12.5	12.7	14.5
	A/swine/Gent/243/2018_H1N1_Clade_1C.2.1HA	3.3	3.9	1.4	2.2	9.4	10.7	10.5	11.3	4.8	5.2	5.5	13.4	10.5	11.7	12.9	13.8
	A/swine/Gent/05/2019_H1N1_Clade_1C.2.1HA	6.0	2.2	5.8	6.2	8.7	8.8	8.9	10.1	7.5	7.9	8.0	13.2	9.4	11.1	11.1	13.0
	A/swine/Gent/06/2019_H1N1_Clade_1C.2.1HA	6.3	2.4	6.7	6.8	8.7	8.6	8.7	10.0	8.0	8.3	8.3	13.6	9.7	11.0	11.2	13.3
	A/swine/Gent/09/2019_H1N2_Clade_1C.2.1HA	3.3	3.9	1.7	2.1	8.6	10.0	9.8	10.5	4.2	4.6	5.2	12.5	9.7	10.9	12.1	13.0
	A/swine/Gent/31/2019_H1N1_Clade_1C.2.1HA	5.4	2.9	3.8	5.0	9.2	9.9	9.9	11.0	6.8	7.2	7.7	12.8	9.5	11.8	11.4	12.8
	A/swine/Gent/54/2019_H1N2_Clade_1C.2.1HA	6.3	3.6	4.3	5.8	9.3	9.9	10.0	11.1	7.4	7.8	8.4	12.4	9.0	12.1	10.9	12.1
	A/swine/Gent/99/2019_H1N1_Clade_1C.2.1HA	5.3	2.8	3.8	4.9	9.2	9.8	9.9	11.0	6.8	7.2	7.6	12.9	9.6	11.8	11.5	12.3
	A/swine/Gent/124/2019_H1N2_Clade_1C.2.1HA	6.2	3.8	3.9	5.5	9.4	10.0	10.1	11.2	7.2	7.6	8.2	12.2	9.0	12.1	10.9	12.9
	A/swine/Gent/205/2019_H1N1_Clade_1C.2.1HA	7.0	3.8	5.6	6.7	8.8	9.0	9.2	10.4	7.9	8.3	8.9	11.8	8.2	11.5	9.8	11.5
	A/swine/Gent/208/2019_H1N1_Clade_1C.2.1HA	6.8	3.6	5.3	6.6	9.6	9.9	10.1	11.2	8.1	8.5	9.0	12.9	9.4	12.3	11.0	12.7
	A/swine/Gent/236/2019_H1N1_Clade_1C.2.1HA	5.7	2.9	4.6	5.7	10.1	10.5	10.6	11.7	7.7	8.1	8.2	14.2	10.7	12.6	12.5	14.2
	A/swine/Netherlands/Gent_157/2017_H1N1_Clade1C.2.2HA	1.5	4.0	3.0	0.7	8.8	10.2	9.9	10.6	3.5	3.8	3.8	13.8	10.9	10.7	13.4	14.4
	A/swine/Netherlands/Gent_31/2018_H1N1_Clade1C.2.2HA	1.8	3.6	2.7	1.2	8.8	10.1	9.9	10.6	3.9	4.3	4.2	13.8	10.8	10.9	13.2	14.2
	A/swine/Netherlands/Gent_32/2018_H1N1_Clade1C.2.2HA	1.1	4.1	3.8	0.9	7.9	9.4	9.1	9.6	2.6	2.9	2.8	13.3	10.5	9.7	12.9	13.9
	A/swine/Netherlands/Gent_103/2018_H1N1-Clade_1C.2.2HA	2.1	4.1	2.4	0.9	8.9	10.4	10.1	10.8	3.8	4.1	4.3	13.6	10.8	11.0	13.2	14.2
	A/swine/Gent/29/2019_H1N1_Clade_1C.2.2HA	5.2	1.7	5.0	5.3	8.2	8.6	8.7	9.8	6.6	7.0	7.2	12.7	9.1	10.7	10.9	12.6
	A/swine/Gent/184/2019_H1N1_Clade_1C.2.2HA	6.2	3.3	4.5	5.8	9.3	9.8	9.9	11.0	7.4	7.6	8.3	12.6	9.2	12.0	11.0	12.2
	A/swine/Netherlands/Gent_193/2019_H1N1_1C.2.2HA	4.3	4.3	3.5	3.3	6.9	8.4	8.2	8.9	3.7	7.8	5.3	10.5	7.6	9.5	10.1	12.5
	A/swine/Gent/202/2019_H1N1_Clade_1C.2.2HA	6.3	2.9	5.3	6.1	8.4	8.7	8.9	10.1	7.3	4.1	8.2	12.1	8.5	11.1	10.2	10.9
	A/swine/Gent/203/2019_H1N1_Clade_1C.2.2HA	5.1	2.4	4.3	4.8	7.6	8.3	8.3	9.4	5.9	7.7	6.9	11.6	8.1	10.3	10.1	11.9
	A/swine/Gent/204/2019_H1N1_Clade_1C.2.2HA	6.1	2.9	5.0	5.8	8.5	8.9	9.0	10.1	7.1	6.3	8.0	12.1	8.5	11.1	10.3	11.6
	A/swine/Gent/30/2014_H1N2_Clade_1B.1.2.1HA	11.4	9.7	13.6	12.0	4.6	2.2	2.7	3.3	10.3	10.4	10.7	10.6	7.6	4.7	7.9	10.4
	A/swine/Gent/35/2014_H1N2_Clade_1B.1.2.1HA	11.6	9.7	13.5	12.1	4.7	2.3	2.9	3.6	10.4	10.5	10.9	10.3	7.2	5.1	7.4	10.0
	A/swine/Gent/93/2014_H1N2	10.3	8.9	12.5	10.8	3.2	1.0	1.2	1.9	9.0	9.0	9.4	10.0	7.0	3.5	7.7	10.0
	A/swine/Gent/94/2014_H1N2_Clade_1B.1.2.1HA	10.2	9.7	12.1	10.3	2.0	2.9	2.4	1.5	7.9	7.9	8.7	8.2	6.1	2.2	7.4	8.7

		Reference viruses															
		EU H1av				EU H1hu				H1pdm09		US H1γ	US H1δ		Human H1		
		**swFI82**	**swBE98**	**swG05**	**swG10**	**swSC94**	**swG99**	**swG02**	**swG12**	**CA09**	**SL15**	**swOH07**	**swIL10**	**swAL16**	**TW86**	**NC99**	**BR07**
	A/swine/Gent/175/2014_H1N2	10.7	9.2	12.8	11.2	3.4	1.2	1.5	2.1	9.3	9.3	9.8	9.8	6.8	3.7	7.5	9.7
	A/swine/Gent/36/2016_H1N2_Clade_1B.1.2.1HA	9.9	9.0	12.1	10.2	2.0	1.6	1.1	0.4	8.0	8.0	8.6	9.2	6.5	2.1	11.5	9.5
	A/swine/Gent/5/2017_H1N2_Clade1B.1.2.1HA	10.0	9.2	12.3	10.4	2.2	1.6	1.2	0.4	8.3	8.2	8.8	9.3	6.7	2.1	7.7	9.6
	A/swine/Netherlands/Gent_7/2018_H1N2_Clade1B.1.2.1HA	14.3	14.2	16.2	14.3	6.5	6.9	6.6	5.4	11.6	11.5	12.4	8.8	8.6	5.0	7.8	9.6
	A/swine/Netherlands/Gent_9-p18/2018_H1N2_Clade1B.1.2.1HA	11.3	9.3	12.7	11.6	4.3	2.4	3.0	3.9	10.0	10.1	10.9	8.4	5.2	5.6	9.1	8.0
	A/swine/Gent/77/2018_H1N2_Clade_1B.1.2.1HA	9.8	8.4	12.4	10.5	3.8	1.9	2.0	2.7	8.9	8.9	9.0	11.3	8.1	3.9	5.4	11.3
	A/swine/Gent/180/2018_H1N2_Clade_1B.1.2.1HA	9.6	8.3	12.0	10.2	2.9	1.1	1.1	1.9	8.3	8.4	8.7	10.4	7.3	3.3	8.9	10.5
	A/swine/Netherlands/Gent_185/2018_H1N2_Clade_1B.1.2.1HA	10.5	8.6	12.7	11.2	4.4	2.0	2.5	3.4	9.6	9.7	10.0	11.0	7.7	4.9	8.2	10.8
	A/swine/Gent/189/2018_H1N2_1B.1.2.1HA	12.0	8.9	13.0	12.5	7.6	5.5	6.2	7.4	11.8	12.0	12.4	11.4	7.7	6.1	8.2	10.5
	A/swine/Gent/216/2018_H1N2_Clade_1B.1.2.1	11.2	8.7	12.3	11.4	5.0	3.1	3.8	4.9	10.2	10.3	11.0	8.9	5.4	6.6	7.4	8.4
	A/swine/Gent/233/2018_H1N2_Clade_1B.1.2.1HA	11.4	11.3	13.1	11.3	3.8	5.0	4.5	3.5	8.7	8.6	9.6	7.6	6.6	3.3	5.5	8.4
	A/swine/Gent/28/2019_H1N2_Clade_1B.1.2.1HA	9.6	9.2	11.5	9.7	1.5	2.9	2.4	1.7	7.3	7.2	8.1	8.3	6.0	2.2	7.8	8.8
	A/swine/Gent/56/2019_H1N2_Clade_1B.1.2.1HA	10.2	8.6	12.1	10.6	3.0	0.6	1.3	2.3	8.8	8.9	9.5	9.3	6.2	4.0	7.5	9.3
	A/swine/Gent/76/2019_H1N2_Clade_1B.1.2.1HA	10.7	8.8	12.5	11.1	3.9	1.5	2.2	3.3	9.5	9.6	10.2	9.6	6.3	4.9	6.9	9.3
	A/swine/Gent/114/2019_H1N2_Clade_1B.1.2.1HA	10.4	8.9	12.7	11.0	3.6	1.3	1.7	2.4	9.2	9.3	9.6	10.4	7.3	3.8	6.7	10.4
	A/Swine/Gent/119/2019_H1N2_Clade_1B.1.2.1HA	10.2	9.0	12.3	10.6	2.6	0.9	0.9	1.4	8.7	8.7	9.2	9.4	6.5	3.1	7.4	9.5
	A/swine/Gent/178/2019_H1N2_Clade_1B.1.2.1HA	9.9	8.5	12.0	10.4	2.6	0.4	0.8	1.8	8.5	8.6	9.1	9.4	6.3	3.5	7.2	9.5
	A/swine/Gent/180/2019_H1N2_Clade_1B.1.2.1HA	10.1	8.7	12.2	10.5	2.7	0.6	0.8	1.7	8.6	8.7	9.2	9.5	6.5	3.4	7.3	9.5
	A/swine/Gent/206/2019_H1N2_Clade_1B.1.2.1HA	10.4	9.9	12.2	10.5	2.2	3.1	2.7	2.0	8.1	8.0	9.0	7.7	5.7	2.6	7.0	8.3
	A/Swine/Gent/228/2014_H1N2_Clade_1A.3.3.2HA	9.0	10.9	9.8	8.2	7.4	9.8	9.3	8.8	5.8	5.6	7.2	9.4	9.0	8.3	11.5	10.9
	A/swine/Gent/121/2018_H1N1_Clade_1A.3.3.2HA	3.2	6.0	5.2	2.6	7.1	9.1	8.7	8.9	0.9	1.0	2.2	12.3	9.9	8.7	12.5	13.1
	A/swine/Gent/53/2019_H1N1_Clade_1A.3.3.2HA	4.4	6.5	4.9	3.2	7.2	9.3	8.9	9.2	1.9	2.1	3.8	11.1	9.1	9.1	11.7	12.1
	A/swine/Gent/220/2019_H1N2_Clade_1A.3.3.2HA	7.5	9.4	6.9	6.2	10.0	10.7	10.3	10.3	4.8	4.9	6.6	9.9	9.0	10.1	11.6	11.1
	A/swine/Gent/235/2019_H1N2_Clade_1A.3.3.2HA	13.3	9.9	12.3	13.0	10.1	9.0	9.6	10.8	12.9	13.3	14.2	9.7	6.7	12.4	6.0	8.5
