# Supplementary material for: Genetic and antigenic evolution of H1 swine influenza A viruses isolated in Belgium and the Netherlands from 2014 through 2019
Source: Sci Rep. 2021 May 28;11:11276. doi: 10.1038/s41598-021-90512-z (PMC8163766; doi:10.1038/s41598-021-90512-z)
Supplement: Supplementary file 4 — Supplementary Information 4. [file 41598_2021_90512_MOESM4_ESM.pdf]

Genetic and antigenic evolution of H1 swine influenza A viruses isolated in Belgium and the Netherlands from 2014 through 2019

Sharon Chepkwony, Anna Parys, Elien Vandoorn, Wojciech Stadejek, Jiexiong Xie, Jacqueline King, Annika Graaf, Anne Pohlmann, Martin Beer, Timm Harder and Kristien Van Reeth

Supplementary Table S5. Percentage amino acid identities in the 50 antigenic sites between the virus isolates and the reference viruses

		Reference viruses															
		EU H1av				EU H1hu				US H1 delta		Human H1			H1pdm09		US H1γ
		swFI82	swBE98	swG05	swG10	swSC94	swG99	swG02	swG12	swIL10	swAL16	TW86	NC99	BR07	CA09	SL15	OH07
Reference viruses	A/swine/Finistere/2899/1982_H1N1_Clade 1C.1HA																
	A/swine/Belgium/1/1998_H1N1_Clade 1C.2HA	88															
	A/swine/Gent/132/2005_H1N1_Clade 1C.2.1HA	86	94														
	A/swine/Gent/28/2010_H1N1_Clade 1C.2.1HA	82	90	96													
	A/swine/Scotland/410440/1994_H1N2_Clade 1B.1HA	40	48	46	46												
	A/swine/Gent/7625/1999_H1N2_Clade 1B.1.2.1HA	40	46	44	40	88											
	A/swine/Gent/177/2002_H1N2_Clade 1B.1.2.1HA	36	42	40	40	92	96										
	A/swine/Gent/26/2012_H1N2_Clade 1B.1.2.1HA	36	42	40	38	86	94	94									
	A/swine/Illinois/A01047020/2010_H1N2_Clade 1B.2.2.2HA	42	46	46	46	74	66	70	66								
	A/swine/Alabama/A01104091/2016_H1N2_Clade 1B.2.2.1HA	44	50	50	48	70	66	66	66	90							
	A/Taiwan/1/1986_H1N1_Clade Other human 1B.2HA	44	50	48	44	82	88	84	82	72	76						
	A/New Caledonia/20/1999_H1N1_Clade Other human 1B.2HA	42	48	46	46	76	72	76	70	84	84	82					
	A/Brisbane/59/2007_H1N1_Clade Other human 1B.2HA	42	48	46	46	74	66	70	64	84	80	76	94				
	A/California/04/2009_H1N1_Clade 1A.3.3.2HA	70	72	70	68	46	42	42	44	50	54	46	48	48			
	A/Slovenia/2903/2015_H1N1_Clade 1A.3.3.2HA	66	66	64	62	40	36	36	38	44	48	40	42	42	92		
	A/swine/Ohio/511445/2007_H1N1_Clade 1A.3.3.3HA	64	66	66	64	46	42	42	42	46	46	46	48	48	88	84	
Virus isolates	A/swine/Netherlands/Gent_05/2014_H1N1_Clade 1C.2.1HA	80	86	90	90	46	42	40	40	42	46	46	42	42	64	58	60
	A/swine/Gent/16/2014_H1N1_Clade 1C.2.1HA	80	88	94	94	46	42	40	40	46	50	46	46	46	66	60	62
	A/swine/Gent/36/2014_H1N1_Clade 1C.2.1HA	76	82	88	88	42	38	36	36	42	46	42	42	42	62	58	60
	A/swine/Netherlands/Gent_46/2014_H1N1_Clade 1C.2.1HA	82	88	92	92	44	40	38	38	44	48	44	44	44	66	60	62
	A/swine/Netherlands/Gent_55/2014_H1N1_Clade 1C.2.1HA	74	82	88	88	44	40	38	38	44	48	44	42	42	62	58	60
	A/swine/Gent/61/2014_H1N1_Clade 1C.2.1HA	74	82	86	86	42	38	36	36	44	48	44	44	44	62	58	62
	A/swine/Gent/78/2014_H1N1_Clade 1C.2.1HA	80	88	92	92	46	42	40	40	48	50	48	48	48	66	60	64
	A/swine/Gent/121/2014_H1N1_Clade 1C.2.1HA	82	90	96	96	48	44	42	42	48	52	48	46	46	70	64	66
	A/swine/Gent/122/2014_H1N1_Clade 1C.2.1HA	82	90	96	96	48	44	42	42	48	52	48	46	46	70	64	66
	A/swine/Gent/48/2015_H1N1_Clade 1C.2.1HA	78	82	88	88	42	38	36	36	44	48	42	44	44	64	60	66
	A/swine/Netherlands/Gent_62/2015_H1N1_Clade 1C.2.1HA	78	86	92	92	44	40	38	40	48	50	46	46	46	66	60	62
	A/swine/Gent/173/2015_H1N1_Clade 1C.2.1HA	76	86	88	88	44	40	38	38	44	48	44	44	44	66	60	68
	A/swine/Gent/150/2016_H1N1_Clade 1C.2.1HA	80	88	94	94	48	44	42	42	48	52	48	46	46	68	62	64
	A/swine/Gent/13/2017_H1N1_Clade1C.2.1HA	80	88	94	94	46	42	40	40	46	50	46	44	44	72	66	68

		Reference viruses																	
		EU H1av				EU H1hu				US H1 delta		Human H1			H1pdm09		US H1γ
		swFI82	swBE98	swG05	swG10	swSC94	swG99	swG02	swG12	swIL10	swAL16	TW86	NC99	BR07	CA09	SL15	OH07
	A/swine/Gent/121/2017_H1avN2_Clade1C.2.1HA	72	80	84	84	40	36	34	34	42	46	40	42	42	62	62	64
	A/swine/Gent/138/2017_H1N1_Clade1C.2.1HA	78	84	86	86	44	40	38	38	48	50	44	46	46	64	58	64
	A/swine/Gent/180/2017_H1N1_Clade1C.2.1HA	76	84	88	88	44	40	38	38	46	50	44	46	46	66	60	64
	A/swine/Netherlands/Gent_8/2018_H1N1_Clade1C.2.1HA	76	80	86	86	42	38	36	36	44	48	42	44	44	62	58	64
	A/swine/Netherlands/Gent_9-p20/2018_H1N1_Clade 1C.2.1HA	70	78	82	82	40	38	36	36	42	46	44	42	42	62	58	60
	A/swine/Gent/127/2018_H1N1_Clade 1C.2.1HA	80	88	94	94	46	42	40	40	46	50	46	44	44	68	64	64
	A/swine/Gent/196/2018_H1N1_Clade 1C.2.1HA	74	82	88	88	44	42	40	40	44	48	48	42	42	62	58	60
	A/swine/Gent/241/2018_H1N1_Clade 1C.2.1HA	74	84	86	86	44	40	38	38	44	48	44	44	44	64	58	66
	A/swine/Gent/243/2018_H1N1_Clade 1C.2.1HA	76	84	90	90	44	40	38	38	46	50	44	44	44	70	68	70
	A/swine/Gent/05/2019_H1N1_Clade 1C.2.1HA	74	80	86	86	42	42	40	40	42	46	46	44	44	62	56	64
	A/swine/Gent/06/2019_H1N1_Clade 1C.2.1HA	76	82	88	88	44	44	42	42	44	48	48	46	46	64	58	66
	A/swine/Gent/09/2019_H1N2_Clade 1C.2.1HA	76	84	90	90	44	40	38	38	46	50	44	44	44	70	68	70
	A/swine/Gent/31/2019_H1N1_Clade 1C.2.1HA	76	84	90	90	44	40	38	38	46	50	44	44	44	70	68	70
	A/swine/Gent/54/2019_H1N2_Clade 1C.2.1HA	76	86	88	88	42	38	36	36	42	46	42	40	40	68	68	68
	A/swine/Gent/99/2019_H1N1_Clade 1C.2.1HA	76	84	90	90	42	38	36	36	42	46	42	40	40	68	68	68
	A/swine/Gent/124/2019_H1N2_Clade 1C.2.1HA	74	82	88	88	44	40	38	38	46	50	44	44	44	70	68	70
	A/swine/Gent/205/2019_H1N1_Clade 1C.2.1HA	74	82	88	88	44	40	38	38	44	48	42	42	42	68	66	68
	A/swine/Gent/208/2019_H1N1_Clade 1C.2.1HA	78	86	92	92	46	42	40	40	46	50	46	44	44	66	60	66
	A/swine/Gent/236/2019_H1N1_Clade 1C.2.1HA	80	88	94	94	48	44	42	42	48	52	48	46	46	68	62	64
	A/swine/Netherlands/Gent_157/2017_H1N1_Clade1C.2.2HA	82	90	96	96	44	40	38	38	44	48	44	44	44	68	64	66
	A/swine/Netherlands/Gent_31/2018_H1N1_Clade1C.2.2HA	80	88	94	94	44	40	38	38	42	46	42	42	42	66	62	64
	A/swine/Netherlands/Gent_32/2018_H1N1_Clade1C.2.2HA	80	88	94	94	44	40	38	38	44	48	44	44	44	68	64	66
	A/swine/Netherlands/Gent_103/2018_H1N1-Clade 1C.2.2HA	82	90	96	96	44	40	38	38	44	48	44	44	44	68	64	66
	A/swine/Gent/29/2019_H1N1_Clade 1C.2.2HA	76	84	88	88	42	38	36	36	40	44	40	40	40	64	60	66
	A/swine/Gent/184/2019_H1N1_Clade 1C.2.2HA	76	84	88	88	42	38	36	36	40	44	40	40	40	64	60	66
	A/swine/Netherlands/Gent_193/2019_H1N1_1C.2.2HA	76	84	86	86	42	38	36	36	40	44	40	40	40	66	62	64
	A/swine/Gent/202/2019_H1N1_Clade 1C.2.2HA	76	84	88	88	42	38	36	36	40	44	40	40	40	64	60	66
	A/swine/Gent/203/2019_H1N1_Clade 1C.2.2HA	76	84	88	88	42	38	36	36	40	44	40	40	40	64	60	66
	A/swine/Gent/204/2019_H1N1_Clade 1C.2.2HA	76	84	88	88	42	38	36	36	40	44	40	40	40	64	60	66
	A/swine/Gent/30/2014_H1N2_Clade 1B.1.2.1HA	36	42	40	38	84	90	90	92	66	66	82	68	64	44	38	42
	A/swine/Gent/35/2014_H1N2_Clade 1B.1.2.1HA	36	42	40	38	86	92	92	94	68	68	82	70	66	44	38	42
	A/swine/Gent/94/2014_H1N2_Clade 1B.1.2.1HA	38	40	40	38	86	92	92	94	70	68	82	70	64	44	38	42
	A/swine/Gent/36/2016_H1N2_Clade 1B.1.2.1HA	36	42	40	38	78	86	84	82	62	64	80	68	62	40	34	38
	A/swine/Gent/5/2017_H1N2_Clade1B.1.2.1HA	36	42	40	38	78	86	84	82	62	64	80	68	62	40	34	38
	A/swine/Netherlands/Gent_7/2018_H1N2_Clade1B.1.2.1HA	40	44	42	40	72	78	76	74	58	62	76	66	62	42	36	40
	A/swine/Netherlands/Gent_9-p18/2018_H1N2_Clade1B.1.2.1HA	36	42	40	38	80	84	82	80	60	62	78	66	64	40	34	38
	A/swine/Gent/77/2018_H1N2_Clade 1B.1.2.1HA	36	42	40	38	78	86	84	82	62	64	80	68	62	40	34	38
	A/swine/Gent/180/2018_H1N2_Clade 1B.1.2.1HA	36	42	40	38	78	86	84	82	62	64	80	68	62	40	34	38

		Reference viruses															
		EU H1av				EU H1hu				US H1 delta		Human H1			H1pdm09		US H1γ
		swFI82	swBE98	swG05	swG10	swSC94	swG99	swG02	swG12	swIL10	swAL16	TW86	NC99	BR07	CA09	SL15	OH07
	A/swine/Netherlands/Gent_185/2018_H1N2_Clade 1B.1.2.1HA	36	42	40	38	76	84	82	80	60	62	78	66	60	40	34	38
	A/swine/Gent/189/2018_H1N2_Clade 1B.1.2.1HA	36	42	40	38	74	82	80	78	62	64	76	64	60	40	34	38
	A/swine/Gent/216/2018_H1N2_Clade 1B.1.2.1	36	42	40	38	78	86	84	82	62	64	80	68	62	40	34	38
	A/swine/Gent/233/2018_H1N2_Clade 1B.1.2.1HA	40	42	42	40	76	84	82	80	64	66	78	66	60	42	36	38
	A/swine/Gent/28/2019_H1N2_Clade 1B.1.2.1HA	38	40	40	38	74	82	80	78	62	64	76	64	58	40	34	36
	A/swine/Gent/56/2019_H1N2_Clade 1B.1.2.1HA	36	42	40	38	76	84	82	80	64	66	78	70	64	40	34	38
	A/swine/Gent/76/2019_H1N2_Clade 1B.1.2.1HA	38	40	38	36	74	82	80	78	58	60	76	64	58	38	32	36
	A/swine/Gent/114/2019_H1N2_Clade 1B.1.2.1HA	36	42	40	38	78	86	84	82	62	64	80	68	62	40	34	38
	A/Swine/Gent/119/2019_H1N2_Clade 1B.1.2.1HA	36	42	40	38	78	86	84	82	62	64	80	68	62	40	34	38
	A/swine/Gent/178/2019_H1N2_Clade 1B.1.2.1HA	34	40	38	36	76	84	82	80	60	62	78	66	60	38	32	36
	A/swine/Gent/180/2019_H1N2_Clade 1B.1.2.1HA	34	40	38	36	76	84	82	80	60	62	78	66	60	38	32	36
	A/swine/Gent/206/2019_H1N2_Clade 1B.1.2.1HA	38	40	40	38	74	82	80	78	66	62	76	64	62	40	34	38
	A/Swine/Gent/228/2014_H1N2_Clade 1A.3.3.2HA	66	68	64	64	46	40	42	42	50	50	44	48	46	88	86	78
	A/swine/Gent/121/2018_H1N1_Clade 1A.3.3.2HA	64	64	62	60	36	32	32	34	40	44	36	38	38	84	92	76
	A/swine/Gent/53/2019_H1N1_Clade 1A.3.3.2HA	62	64	62	60	40	36	36	38	44	48	40	42	42	88	94	80
	A/swine/Gent/220/2019_H1N2_Clade 1A.3.3.2HA	62	66	64	64	44	38	40	40	44	46	40	44	42	84	82	72
	A/swine/Gent/235/2019_H1N2_Clade 1A.3.3.2HA	52	58	54	54	42	36	38	34	42	42	38	46	46	68	72	66
