# Supplementary material for: Genetic and antigenic evolution of H1 swine influenza A viruses isolated in Belgium and the Netherlands from 2014 through 2019
Source: Sci Rep. 2021 May 28;11:11276. doi: 10.1038/s41598-021-90512-z (PMC8163766; doi:10.1038/s41598-021-90512-z)
Supplement: Supplementary file 5 — Supplementary Information 5. [file 41598_2021_90512_MOESM5_ESM.pdf]

Genetic and antigenic evolution of H1 swine influenza A viruses isolated in Belgium and the Netherlands from 2014 through 2019

Sharon Chepkwony, Anna Parys, Elien Vandoorn, Wojciech Stadejek, Jiexiong Xie, Jacqueline King, Annika Graaf, Anne Pohlmann, Martin Beer, Timm Harder and Kristien Van Reeth

Supplementary Table S6: Neuramidase inhibition (NI) antibody titers against reference viruses used in preliminary subtyping.

Virus isolate	NI antibody titers against:				Isolate subtype
	swBE98 (H1avN1)	CA09 (H1pdm09N1)	swG99 (H1N2)	swFL98 (H3N2)	
A/swine/Gent/34/2014	<160	<160	<160	640	H3N2
A/swine/Gent/175/2014	<160	320	20480	640	H1N2

Full virus names: swBE98 - A/swine/Belgium/1/1998; CA09 - A/California/04/2009; swG99 - A/swine/Gent/7625/1999; swFL98 - A/swine/Flanders/1/1998
